# Abiotic Factors Modulating Metabolite Composition in Brown Algae (Phaeophyceae): Ecological Impacts and Opportunities for Bioprospecting of Bioactive Compounds

**DOI:** 10.3390/md22120544

**Published:** 2024-12-02

**Authors:** Clara Lopes, Johana Marcela Concha Obando, Thalisia Cunha dos Santos, Diana Negrão Cavalcanti, Valéria Laneuville Teixeira

**Affiliations:** 1 Laboratory of Natural Products from Seaweeds (ALGAMAR), Department of Marine Biology, Institute of Biology, Federal Fluminense University, Niterói 24210-201, RJ, Brazil; lopes_clara@id.uff.br (C.L.); mc1226@gmail.com (J.M.C.O.); thalisiacunhaq@gmail.com (T.C.d.S.); valerialaneuville@gmail.com (V.L.T.); 2 Ideas Aquarium, Scientific and Technological Base Incubator of the Ribeira Valley and South Coast of São Paulo, São Paulo State University “Júlio de Mesquita Filho”, Registro 11900-000, SP, Brazil; 3 National Institute of Science and Technology in Nanotechnology for Sustainable Agriculture, INCTNanoAgro, Sorocaba 18087-180, SP, Brazil; 4 Postgraduate Program in Marine Biology and Coastal Ecosystems, Institute of Biology, Federal Fluminense University, Niterói 24210-201, RJ, Brazil; 5 Postgraduate Program in Neotropical Biodiversity, Institute of Biosciences, Federal University of the State of Rio de Janeiro, Rio de Janeiro 22290-240, RJ, Brazil; 6 Postgraduate Program in Science and Biotechnology, Institute of Biology, Federal Fluminense University, Niterói 24210-201, RJ, Brazil

**Keywords:** brown seaweed, Phaeophyceae, natural products, abiotic parameters

## Abstract

Brown algae are vital structural elements and contributors to biodiversity in marine ecosystems. These organisms adapt to various environmental challenges by producing primary and secondary metabolites crucial for their survival, defense, and resilience. Besides their ecological role, these diverse metabolites have potential for biotechnological applications in industries including pharmaceuticals, cosmetics, and food. A literature review was conducted encompassing studies from 2014–2024, evaluating the effects of hydrodynamics, temperature, light, nutrients, seasonality, and salinity on the chemical profiles of various Phaeophyceae algae species. Thirty original articles spanning 69 species from the Sargassaceae, Dictyotaceae, Fucaceae, and Scytosiphonaceae families were analyzed and systematically arranged, with a focus on methodologies and key findings. This review furthers ecological discussions on each environmental factor and explores the biotechnological potential of metabolites such as polysaccharides, fatty acids, phenolics, diterpenes, and pigments. The information in this work is beneficial for metabolite bioprospecting and in vitro cultivation models as well as indoor and outdoor cultivation studies.

## 1. Introduction

Seaweeds are photosynthetic organisms that are heterogeneous and benthic. They are categorized into three major taxonomic groups based on their pigment profile and morphological, anatomical, and reproductive structures. These groups include Chlorophyta (green algae), Phaeophyceae (brown algae), and Rhodophyta (red algae) [1,2]. They offer numerous ecosystem services and provide various benefits to humans. For instance, algae furnish regulatory services by helping balance the planet’s oxygenic atmosphere through carbon dioxide capture and the production of atmospheric oxygen and some carbon-based molecules. They also provide supportive services by helping to maintain ecosystems through nutrient cycling and offer provisions by providing material consumable by other living beings [3]. Specifically, brown seaweeds significantly contribute to these three categories of ecosystem services that are crucial for the health and sustainability of both coastal ecosystems and human societies.

In recent years, macroalgae have garnered increasing interest from various fields, including fuels, plastics, cosmetics, pharmaceuticals, agrochemicals, energy compounds, and foods [4]. The chemical diversity of macroalgae presents the potential to discover a wide spectrum of primary and secondary metabolites, with interesting properties and applications [5,6].

The main secondary metabolites of algae include lipids, proteins, and polysaccharides, while other substances, such as phenolic compounds, halogenated compounds, sterols, terpenes, and short peptides, constitute the secondary metabolites produced in algal tissues [2,7]. In marine environments, characterized by their complexity and high competitiveness, biological interactions and abiotic factors such as light, temperature, hydrodynamics, and nutrient availability profoundly influence the production of secondary metabolites [8]. These substances play vital roles in the adaptation and evolution of marine organisms, serving key ecological functions in coping with environmental pressures like defense against predators, allelopathy, spatial competition, and regulation of symbiosis. The structural diversity and unique modes of action against the biological targets of these secondary metabolites indicate their potential use in several biotechnological applications in the chemical, pharmaceutical, cosmetic, and nutraceutical industries [9,10,11]. Seaweed research driven by chemical ecology in marine environments underscores the potential therapeutic uses of these metabolites and their importance in drug development while emphasizing their role in population structure, community organization, and ecosystem functioning [12].

Brown algae are the most studied taxonomic group among seaweeds in regards to both primary and secondary metabolism chemistry. This includes substances ranging from well-known polysaccharides like alginates and fucans to fatty acids, sterols, carotenoids, polyphenols (phlorotannins), volatile hydrocarbons, and the unique terpenes with diverse biological activities [11,13,14,15,16]. Among brown algae, several families notably produce significant amounts of secondary metabolites, such as Dictyotaceae, Fucaceae, Sargassaceae, and Laminariaceae [17]. In particular, the Dictyotaceae family is recognized for containing a wide diversity of biologically active compounds [2,18,19,20,21,22,23,24]. Many studies have demonstrated that genera such as *Dictyota*, *Padina*, *Lobophora*, and *Canistrocarpus*, which belong to this family, are especially rich in secondary metabolites with pharmacological and biotechnological potentials [14,16,25].

Over time, numerous studies have concentrated on both the ecological aspects and the identification of natural products derived from brown algae for biotechnological applications. These studies aim to assess how various biological factors influence these algae’s chemical profiles. The purpose of this review is to gather, organize, and offer insights into how different non-biological factors—including hydrodynamics, temperature, light, nutrients, seasonality, and salinity—affect the composition and production of natural products in different brown algal species. Additionally, we discuss the significance of understanding these parameters for bioprospecting metabolites with biotechnological interest.

## 2. Abiotic Ecological Parameters and Brown Seaweed

**Figure 1:**
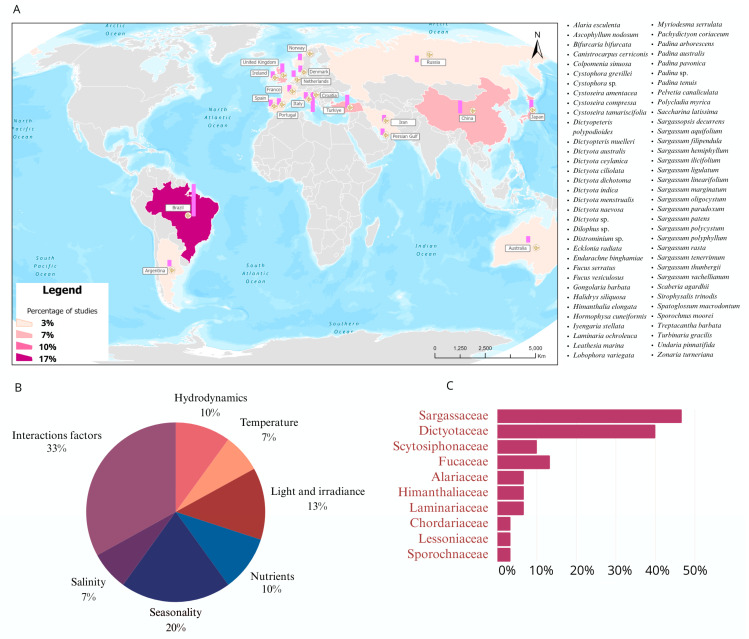
Visual summary of reviewed studies. (**A**) The global distribution of studies evaluating the effects of abiotic parameters on the metabolic profiles of marine macroalgae (to the right of the map, the 69 reviewed macroalgae species are listed); (**B**) pie chart of the percentage of studies focusing on specific abiotic parameters; (**C**) bar graph of the percentage of explored families in these studies.

The search and selection of articles resulted in the inclusion of 30 studies that addressed one or more abiotic factors influencing the chemical composition of brown algae. Among the studies investigating the influence of individual parameters, seasonality was the most researched (20%), followed by light and irradiance (13%), then nutrients and hydrodynamics (10% each), and temperature and salinity (7% each) (Figure 1A). Moreover, many articles (33%) discussed the relationship between two or more factors and the metabolite profile of brown algae.

In total, there are 69 species of brown algae belonging to families such as Sargassaceae (47%), Dictyotaceae (40%), Fucaceae (13%), and Scytosiphonaceae (10%) (Figure 1B). It is important to highlight that species from these families have been extensively examined for their chemical composition and biotechnological potential [18]. With regard to the active components of brown seaweeds in extracts, many studies have primarily focused on evaluating the content of phenolic compounds, terpenoids (including diterpenoids and sterols), pigments (such as chlorophylls and carotenoids), and fatty acids. The influence of abiotic factors on the production and content of these compounds will be discussed in the subsequent sections.

The studies included in this review cover brown algae from the following countries: Brazil (17%), Croatia (10%), China (7%), Japan (7%), Turkey (7%), and the United Kingdom (7%), among others (Figure 1). These percentages indicate that there is no particular country predominating in research on how abiotic factors influence the chemical profiles of brown marine macroalgae species. Despite a well-established understanding of these factors influences, this research area seems to present difficulties in terms of reproducing and manipulating environmental variables both in laboratories and in the field.

### 2.1. Hydrodynamics

Investigation of phenomena such as tides, waves, and currents falls under the purview of hydrodynamics, which pertains to the movement of water in oceans and seas. Hydrodynamics plays a crucial role in determining the behaviors of macroalgae populations by altering nutrient distribution and light absorption, thereby impacting their metabolic processes [26,27]. Several species of brown algae, namely *Canistrocarpus cerviconis* [28], *Lobophora variegata* [28], *Pachydictyon coriaceum* [9], *Pelvetia canaliculata* [29,30], *Ascophyllum nodosum* [29], *Fucus serratus* [29], *Sargassum hemiphyllum* [9], *Sargassum vachellianum* [9], *Endarachne binghamiae* [9], and *Colpomenia sinuosa* [9], have been observed in order to understand how this factor influences their metabolic profile (Table 1). Predominantly, these studies have examined the effect of tidal exposure on the chemical composition of seaweeds, primarily focusing on pigments and phenolic compounds. Some papers have additionally explored the content of proline, thiols, malondialdehyde, hydrogen peroxide, fatty acids, and carbohydrates.

Rodrigues et al. [28] examined the antioxidant activity of three species from the Dictyotaceae family (*C. cerviconis, D. delicatula*, and *L. variegata)* in relation to their exposure to sea environments. The phenolic content for this species, i.e., the algae gathered in Ponta de Pedras (Goiania, Brazil), demonstrated a content of 46.72 ± 1.44 mg GAE/g of extract, and in that from Jaguaribe Beach (Itamaracá Island, Brazil), it was 42.51 ± 1.86 mg GAE/g of extract. They found that the total yield from algae extracted in an exposed intertidal zone at Ponta de Pedras (Goiania, Brazil) was slightly higher compared to that from the submerged intertidal zone at Jaguaribe Beach (Itamaracá Island, Brazil) during low tide. Among the species, *C. cerviconis* exhibited superior antioxidant activity, particularly in the aqueous extract.

Islamova et al. [30] analyzed the biochemical composition of the algae *P. canaliculata* during different phases of the tidal cycle. The intracellular content of phlorotannin peaked during low and full tides (7% and 7.4% dry weight (DW), respectively) but sharply decreased after 2 h of submersion. However, cell wall-associated phlorotannins were more prominent during high tides (approximately 0.7% DW). The observed pigments (chlorophyll A, chlorophyll C, and carotenoids) generally exhibited greater abundance during high and ebb tides. There was a peak in chlorophyll A content at 0.8–0.9 mg.g^−1^ DW during the submerged phases before a nearly 50% decrease during the exposed phases. In contrast, pheophytin levels were the lowest among the analyzed pigments.

On the other hand, the levels of photosynthetic pigments, hydrogen peroxide, proline, malondialdehyde, and thiols were evaluated in three samples representing different stages, such as periods of stress and submersion, for this species [29]. The hydrogen peroxide level in *P. canaliculata* peaked (0.19 µmol.g^−1^ DW) shortly after submersion, then gradually decreased until the ebb tide phase, and sharply declined after the thalli were exposed. This decrease in H_2_O_2_ content coincided with a rise in malondialdehyde levels, which reached a peak of 74 nmol.g^−1^ DW during the low-water phase [30].

The studies examined in this work [28,29,30] suggest that tidal dynamics influence not just nutrient uptake but also the production of bioactive compounds like polyphenols and carotenoids, which are crucial for the ecological interactions and defense mechanisms of algae [31]. For example, phenolic compounds play several ecological roles, including UV protection and deterrence of herbivores. Their concentrations change in response to environmental gradients such as salinity and light exposure. Specifically, areas of higher latitudes and increased salinity demonstrate elevated levels of phenolic compounds, suggesting that hydrodynamic conditions could stimulate their production in reaction to environmental stressors [32].

Similarly, the pigment content, including chlorophylls, fucoxanthin, and β-carotene, is indispensable for photosynthesis and may also contribute to the antioxidant activity essential for cellular function [33]. The interplay between hydrodynamic forces and pigments highlights the adaptive capacity of brown algae in their dynamic marine environments [34]. Therefore, understanding tidal influences is crucial for comprehending the biochemical profiles of these organisms.

### 2.2. Temperature

Temperature significantly impacts the growth, development, and productivity of macroalgae. Additionally, it plays a pivotal role in determining the geographical distribution and reproductive capabilities of seaweeds by influencing their metabolic rates [35]. Among the ten articles that examined the influence of temperature on chemical composition (Table 2), the most studied families of brown algae were Dictyotaceae (50%), Sargassaceae (40%), and Scytosiphonaceae (30%). There were also studies on the families Alariaceae (10%), Chordariaceae (10%), Fucaceae (10%), and Himanthaliaceae (10%). Concerning the temperatures examined in the articles, they covered a range from 5 °C to 60 °C, depending on the specific objectives of the study or the location of the investigation. Among the metabolites investigated, phenolic compounds (70%) and pigments (20%) were the most researched, but there were also studies focusing on diterpenes (10%).

Through varied objectives, the authors analyzed the role of temperature as an abiotic factor that influenced the chemical composition of brown algae. Mekinić et al. [38] scrutinized the biological potential of *D. dichotoma* and *P. pavonica* as well as the impact of the extraction method (ultrasound or solvent) and temperature regime on their phenolic profile. The ethanolic extracts demonstrated higher concentrations of phenolics, and the extraction temperature did not affect the yield. The discussion highlights that even though elevated temperatures generally enhance extraction, many phenolics are heat-sensitive, leading to a decrease in the total amount extracted. In marine environments, high environmental temperatures can negatively impact the production of phenolic compounds in algae due to their thermosensitivity.

Obando et al. [19] studied the optimal cultivation conditions for *D. menstrualis* by analyzing diterpene profiles at both ambient temperature (23.2 ± 0.17 °C) and a consistent laboratory temperature (21 ± 1 °C). After 180 days at the controlled temperature, they observed a yield of 2.10% ± 0.33 as compared to 4.33% ± 0.14 at room temperature, signifying a considerable drop in overall performance under controlled conditions. Despite this, diterpene production increased, with pachydictyol A jumping from 8.79% to 15.10% and 5-acetoxy-1,6-cycloxenia-2,13-diene-16,17-dial moving from 21.98% to 24% in relative abundance. In short-term studies (60 days), they reported a 10–20% increase in diterpene concentrations, especially in acetylated forms, which seem to function as an adaptive response to environmental stress. Although temperature was not the primary focus, the steady 21 °C in the laboratory may have had a significant impact on the diterpene profiles.

The other eight articles that investigated the impact of temperature on the chemical composition of brown algae also explored additional abiotic factors and their interrelationships [9,36,37,39,40,41,42,43]. As a result, a separate section of the present article was created to address these investigations in detail.

Hurd et al. [4] indicated that temperature serves as a key seasonal indicator in mid-to-high latitudes, suggesting that variations in seawater temperature correspond directly to the amount of light that penetrates the water. This highlights the robust relationship between temperature and light, as opposed to considering temperature as an isolated factor [4]. Changes in metabolic pathways may enhance the ability of seaweeds to resist and adapt to stress caused by rising temperatures, indicating that certain algal metabolites play a substantial role in moderating environmental stressors [35].

### 2.3. Light and Irradiance

The term “light” refers to the waves of the electromagnetic spectrum that are visible to the human eye, excluding ultraviolet and infrared wavelengths. “Irradiance” quantifies the amount of light incident on a surface [4]. These factors significantly influence the energy available for photosynthesis, thereby affecting energy supply across the food chain and algae growth rates [4,44]. The interplay between light and brown algae is vital for their growth and biochemical composition. Numerous studies underscore how varied light conditions impact the physiological and chemical responses of brown algae, particularly concerning growth rates, pigment composition, and secondary metabolites [45,46,47].

Nine articles identified in our bibliographic search broadly explored the effects of light and irradiance on the metabolism of brown seaweeds (Table 3). The Sargassaceae family was the most frequently studied (55.56%), followed by Laminariaceae (22%), Alariaceae (11%), and Fucaceae (11%). The types of lights studied varied and included fluorescent light, LED, UVA, UVB, light excluding UV, and natural light, while the irradiance fluctuated between 0 and 502 μmol photons m^−2^ s^−1^. Regarding the most analyzed metabolites, phenolic compounds and pigments were noteworthy, each accounting for 44.44% of the analyses.

Öztaşkent et al. [45] conducted a study to discern the impact of different spectra of LED lights on the growth, biochemical composition, and pigment composition of *T. barbata.* The algal thalli were cultivated under four LED light conditions—red, green, yellow, and blue—with an intensity of 100 μmol photon m^−2^ s^−1^ continuously for 16 days. A control group was also maintained under fluorescent light conditions. They discovered that LED lights significantly affected *T. barbata*’s growth rate, increasing it by 61%. The highest specific growth rate, 2.12  ±  0.09% day^−1^, was observed under red light. The concentrations of all monitored compounds were impacted by the LED lights. Under yellow light, the highest levels of alginates (12.78 ± 0.31%), chlorophyll A (1.30 mg g^−1^ FW), and carotenoids (0.35 mg g^−1^ FW) were reported. On the other hand, exposure to red light resulted in the greatest concentrations of fatty acid methyl esters (FAMEs) (53.74 ± 3.75%) and proteins (15.86 ± 0.94%) content. Finally, green light led to the most substantial levels of chlorophyll C (0.25 mg g^−1^ FW).

The study by Ak et al. [46] aimed to optimize light intensity and salinity for the evaluation of the growth and antioxidant properties of the brown alga *G. barbata.* Utilizing the response surface methodology, a variety of experimental conditions was assessed, culminating in the findings that light and salinity significantly impacted the production of antioxidant compounds. The optimal light intensity of 53.86 µmol photons m^−2^ s^−1^ was found to maximize antioxidant activity without hindering growth, with the highest total phenolic and flavonoid contents being recorded at this level. Moreover, a salinity level of 24.02% was identified as favorable for enhancing antioxidant properties.

Sun et al. [48] compared the responses of *S. thunbergii* under UVB radiation, determining the efficiency of PSII photochemical activities, conducting metabolomic analyses, and measuring the content of major carbon-based metabolites (including soluble sugar, total amino acids, and lipids). The primary metabolites detected were organic acids and amino acids, all of which are intermediates of metabolic processes. In terms of exposure to UVB light treatments, the initial responses emanated from carbon and nitrogen metabolic pathways. The authors further concluded that male macroalgae demonstrated a superior ability to handle the stresses induced by light treatments. This resilience was attributable to the enhanced energy use in their metabolism and the metabolism of their amino acids when exposed to UVB radiation compared to female macroalgae. Thus, male macroalgae exhibited the highest concentration of amino acids when exposed to high UVB light (22.5 μmol.mg prot^−1^), whereas females showed higher levels (17.5 μmol.mg prot^−1^) when exposed to low UVB light.

**Table 3:** The influence of light and irradiance in Phaeophyceae seaweed. In this table, a systematic organization of data from the analyzed articles is presented, documenting only the highest recorded concentrations of metabolites in the column “Representative Content”. Chl: chlorophyll; D: dark; DCM: dichloromethane; DMF: dimethylformamide; DW: dry weight; DWE: dry weight extract; FW: fresh weight; GAE: gallic acid equivalent; L: light; LED: light emitting diode; MeOH: methanol; NR: unregistered; PAR: photosynthetically active radiation; PC: phenolic content; Ref: reference; RGR: relative to growth rate; UV: ultraviolet; W: watts.

Families	Species	Study Area	Photoperiod	Type of Light: Irradiance	Growth Rate	Extract Type	Analyzed Metabolite	Representative Content	Ref
Alariaceae	*Undaria pinnatifida*	Hirota Bay (Japan)	12L:12D	Cool white light without UV: 180 and 30 μmol photons m^−2^ s^−1^	RGR −0.150–0.104 ± 0.657–0.739% day^−1^ (30 μmol photons m^−2^ s^−1^)	Organic extract (DMF)	Pigments: Chl a, Chl c1, Chl c2, fucoxanthin, and violaxanthin + zeaxanthin	Chl a: 0.506 mg.g^−1^ (30 μmol photons m^−2^ s^−1^), ~0.3 mg.g^−1^ (180 μmol photons m^−2^ s^−1^); Chl c1: 0.025 mg.g^−1^ (30 μmol photons m^−2^ s^−1^), ~0.01 mg.g^−1^ (180 μmol photons m^−2^ s^−1^); Chl c2: 0.027 mg.g^−1^ (30 μmol photons m^−2^ s^−1^), ~0.02 mg.g^−1^ (180 μmol photons m^−2^ s^−1^); Fucoxanthin: 0.162 ± 0.008 mg.g^−1^ (30 μmol photons m^−2^ s^−1^), 0.01 mg.g^−1^ (180 μmol photons m^−2^ s^−1^); Violaxanthin + zeaxanthin: 0.062 ± 0.020 mg.g^−1^ (30 μmol photons m^−2^ s^−1^), 0.04 mg.g^−1^ (180 μmol photons m^−2^ s^−1^)	[36]
Fucaceae	*Fucus vesiculosus*	Flaggy Shore, Finavarra, County Clare (Ireland)	12L:12D (7 days)	UVR and PAR: 0–400 mWm^−2^	NR	NR	PC: phlorotannins	510 ± 27.4 μg PGE mg^−1^ DWE	[41]
Laminariaceae	*Saccharina latissima*	Commercial cultivator Zeewaar (The Netherlands)	12L:12D	Cool white fluorescent light: 4.12 ± 0.14 μmol photons m^−2^ s^−1^ and 502.4 ± 3.1 μmol photons m^−2^ s^−1^	NR	DCM extracts	Sterol	Fucosterol: 2.500 mg·kg^−1^ DW; Total sterols: 3.000 mg·kg^−1^ DW	[49]
Laminariaceae	*Saccharina latissima*	Sea farm (Norway)	NR	Natural light: 0–250 μmol photons m^−2^ s^−1^	0.23 ± 0.03 day ^−1^ (high light); 0.66 ± 0.06 mm day ^−1^ (low light)	NR	Protein	77.0 ± 2.7 mg·g^−1^ DW	[50]
Sargassaceae	*Gongolaria barbata*	Turkey	NR	NR: 54 µmol photon m^−2^ s^−1^	NR	NR	Pigments and PC	2.08 mg GAE·g^−1^	[46]
Sargassaceae	*Sargassum filipendula*	Cigarras Beach (north coast of São Paulo State, Brazil)	14L:0D with 3 h of UV exposition during light phase	UVA and UVB: 60 ± 5 μmol photons m^−2^ s^−1^ + UVB and 60 ± 5 μmol photons m^−2^ s^−1^ + UVA	NR	MeOH extract	PC	10.00 ± 0.11 mg GAE.g^−1^ (PAR)	[51]
Sargassaceae	*Sargassum patens*	Unosaki (Japan)	12L:12D	Cool white fluorescent light: 180 μmol photons m^−2^·s^−1^	RGR 6.5% day^−1^ (enriched and 30 °C)	Hidroorganic extract (MeOH:H_2_O, 8:2)	PC: phlorotannins	4.5% DW (upper light)	[40]
Sargassaceae	*Sargassum thunbergii*	Tai Ping Jiao (China)	14L:10D with 8 h of UV exposition during light phase	UV: 150 μmol m^−2^ s^−1^ + 1 W·m^−2^ s^−1^ UVB and 150 μmol m^−2^ s^−1^ + 2.5 + 1 W·m^−2^ s^−1^ UVB	NR	Hidroorganic extract (MeOH/acetonitrile/H_2_O 2:2:1)	Organic acids, depsipeptide, aminoacids, alkaloids, and other classes	Amino acids: 22.5 μmol.mg prot^−1^ (male algae); 17.5 μmol.mg prot^−1^ (female algae)	[51]
Sargassaceae	*Treptacantha barbata*	Kepez District (Çanakkale, Turkey)	24L:0D	LED lights (yellow, blue, green, and red): 100 μmol photon m^−2^ s^−1^	2.12 ± 0.09% day^−1^ (red light)	Aqueous extract (acidified)	Fatty acids, polysaccharides; proteins, pigments	Fatty acids: 53.74 ± 3.75%; 12.78 ± 0.31% (alginate); Proteins: 15.86 ± 0.94%; Chl A: 1.30 mg g^−1^ FW; Chl C: 0.25 mg gr.^−1^ FW; Carotenoids: 0.35 mg gr^−1^ FW	[45]

Polo et al. [51] studied the impact of different radiation treatments (namely control, control + UVA, and control + UVB) conducted over 10 days on the association between potential cellular damage, antioxidant activity, and the bioactive attributes of *S. filipendula* [51]. They discovered a direct connection between phenolic content and antioxidant capability. The extracts without any stress conditions exhibited the highest antioxidant activities and phenolic content (10.00 ± 0.11 mg GAE g^−1^), followed by control + UVA (8.16 ± 0.10 mg GAE g^−1^), and, lastly, control + UVB (7.16 ± 0.09 mg GAE g^−1^). The authors hypothesized that this might be because of the exudation of phenolic compounds that shape a UV light-absorbing environment in the area surrounding it.

Finally, five additional studies investigated the effects of light and irradiance on brown algae, along with other parameters [36,41,43,49,50]. Therefore, these articles are discussed in the section that focuses on the interaction of various abiotic factors.

The impact of varying light spectra and intensities on the growth and biochemical composition of brown algae offers crucial understanding for enhancing future cultivation and bioprospecting of natural products. Studies indicate that specific wavelengths, such as red and yellow light, can considerably boost growth rates and influence the concentrations of compounds like alginates, fatty acids, and chlorophyll. Additionally, optimizing light intensity in conjunction with other parameters, such as salinity, can enhance antioxidant properties. Research concerning UV radiation underscores stress-induced responses in algae, including photoprotective mechanisms and species-specific metabolic alterations, which could be utilized to augment antioxidant production and procure molecules with UV-protective capabilities.

### 2.4. Nutrients

Nutrients are primarily required for the growth and photosynthesis of algae, acting as limiting factors in different physiological processes. Natural sources of these nutrients entail release from sediments, carried by tidal and wind mixing, while non-natural sources include anthropogenic contributions such as fertilizers and pollution [4].

In general, the seven articles identified in our bibliographic search examined nutrient availability and its impact on the metabolic composition of brown algae (Table 4). The families Dictyotaceae and Sargassaceae were the most extensively studied in these articles (30% each), followed by Laminariceae (20%). Other families, such as Alariaceae, Chordariaceae, Scytosiphonaceae, and Fucaceae, were also included in the studies (10% each). The most frequently analyzed nutrients included Provasoli-enriched seawater (PES) (50%), nitrate (30%), and phosphate (20%). The most commonly analyzed parameter was the pigment content (40%).

Obando et al. [52] studied the growth responses and diterpene production of *D. menstrualis* and *C. cerviconis*, maintained in vitro under varied treatment conditions (either in sterilized seawater or in sterilized seawater enriched with Provasoli/2). They observed that the samples exposed to the enriched culture medium demonstrated lower growth rates for both species. For diterpene production, 12 diterpenes in *D. menstrualis* were monitored and shown to correlate positively with the examined parameters (dissolved inorganic nutrients and pH). In contrast, out of ten diterpenes detected in *C. cerviconis* samples, only one showed a positive correlation with abiotic factors, suggesting that diterpene variation could be influenced by other factors. Furthermore, the enriched culture medium appeared favorable for fucosterol production, as this compound displayed increased production levels in the treatment.

Martins et al. [53] also conducted a study on *D. menstrualis*, evaluating the impact of carbon dioxide concentration under limiting conditions and nitrogen saturation on the growth rate, photosynthesis, and biochemical composition of algae. They found that treatments supplemented with NO_3_ resulted in higher growth rates, which remained unaffected by the addition of CO_2_. Furthermore, the biosynthesis of chlorophyll a, proteins, lipids, and polyunsaturated fatty acids was improved in NO_3_^−^-supplemented treatments. The assimilation of nitrate and carbon dioxide was greater in treatments where both NO_3_^−^ and CO_2_ were added, resulting in a noted increase in the photosynthesis rate.

Celis-Plá et al. [54] studied the interactive effects of copper, nitrate, and phosphate treatments on the physiological and metabolic responses of *C. tamariscifolia*. They found that exposure to high levels of copper significantly impacted the algae’s physiology and metabolism. Specifically, the levels of pigment content (such as chlorophyll A and C and fucoxanthin), phenolic compounds, and antioxidant capacity were elevated under high copper conditions, regardless of whether they were combined with phosphate or nitrate-enriched treatments. As for combined photosynthetic capacity, although the changes were not significant, a response to copper exposure was still observed in comparison to other treatments.

The remaining articles that examined how nutrient factors influence the chemical composition of brown algae are discussed in the section on the analysis of abiotic factor interactions, given their coverage of additional parameters [9,36,37,41,43,49,50].

The studies under review shed light on how factors such as nutrient availability, carbon dioxide levels, and exposure to heavy metals can influence the growth, biochemical composition, and metabolite production in brown algae. High nitrogen content in the media might slow down the growth rates but enhance the production of compounds like fucosterol; this can also affect the production of some diterpenes. Although nitrate is known to stimulate certain biochemical responses, other factors like copper can modulate antioxidant capacity and pigment levels. This presents a complex relationship between nutritional conditions and metabolite synthesis, which needs to be highly specific for various taxonomic groups of algae, including on the species level. The findings emphasize the need to optimize nutritional conditions to boost the production of bioactive compounds in brown algae.

The relationship between nutrients and the impact on macroalgae has been studied. It is known that eutrophication stimulates the intense growth of algae in general [4], but studies with *Halimeda* and *Dictyota* spp. seem to have the opposite effect—increased growth with nutrient scarcity. These factors highlight the concern with management practices that harmonize eutrophication control and herbivore preservation, thus ensuring the health and stability of marine ecosystems, such as coral reefs [55].

### 2.5. Salinity

Salinity can influence the marine environment in a variety of ways, including chemical, physical, and biological impacts. The principal effects of salinity involve alterations in osmotic pressure, ion concentrations, and seawater density [4].

The influence of salinity on the chemical composition of brown algae was explored in a total of four articles (Table 5). The Dictyotaceae family was mentioned in all articles that monitored salinity, and the Sargassaceae family was also noted (50%). The most frequently monitored metabolites were phenolic compounds, reaching 88% of the species studied.

Van Heeds et al. [56] evaluated the concentrations of phenolic compounds in algae from various genera, including *Dictyota*, *Dictyopteris, Dilophus, Distromium, Lobophora, Padina, Spatoglossum, Zonaria, Ecklonia, Sargassum, Cystophora, Hormophysa, Myriodesma, Sargassopsis, Sirophysalis, Turbinaria,* and *Sporochnus*. These organisms spanned both temperate and tropical ecosystems along the western coast of Australia. The researchers found that species with higher phenolic concentrations primarily resided in temperate zones, while those in tropical zones had lower phenolic levels. Algae from Eagle Bay (Australia), for instance, had the highest phenolic concentrations, reaching up to 10.99 ± 2.63% dry matter (DM) in *C. grevillei*. In contrast, the lowest concentrations were found in algae from Thevenard Island and Exmouth Gulf (Australia), with a minimum of 0.35 ± 0.03% DM in *D. ceylanica*. Additionally, they found a positive relationship between salinity and phenolic compounds, particularly noticeable in the algae from Shark Bay (Australia). Seaweed species like *S. trinoidis* and *S. decurrens* were more tolerant of higher salinities, potentially accounting for their high phenolic levels in Shark Bay (Australia), which had salinity levels ranging from 39 to 53.5 (1.05 ± 0.34% DM and 0.99 ± 0.12% DM, respectively).

Betancor et al. [57] assessed two sites (S1 and S2) in proximity to a volcanic eruption, comparing them to a control area (S3) with similar physicochemical features [57]. The research aimed to understand whether alterations in physico-chemical parameters influenced the morphology, physiology, and metabolism of the seaweeds *P. pavonica* and *L. variegata*. The areas close to volcanic eruption demonstrated higher salinities than the control location. A noticeable decline in the phenolic compound content of both seaweeds was observed over the semi-diurnal cycles in these areas, whereas the concentration remained stable in the control zone. Moreover, a positive correlation was found between antioxidant activity and phenolic compounds, indicating that seaweeds in areas near the volcanic eruption exhibited reduced antioxidant activity.

Salinity not only influences the quantitative and qualitative chemical composition of brown algae, but it also affects other abiotic conditions, including water pH, ionic concentration, and density, as well as biological factors such as nutrient uptake [4,58,59]. The literature reviewed in this study examined the impact of salinity on the total phenolic content of brown algae. It concluded that higher salinities generally tend to reduce total phenolic levels, except in cases where the algae show a degree of tolerance to elevated salinities, as observed with *S. trinoidis* and *S. decurrens* [56]. Conversely, some studies indicated a correlation between higher concentrations of phenolic compounds and increased salinity [60], while others suggested that lower salinities tend to diminish these concentrations [9,39,58]. Furthermore, the increase in phenolic content observed under reduced salinity conditions may be associated with the intensified toxicity of copper, prompting the algae to activate a defense mechanism by exuding larger quantity of phenols into the surrounding water [58]. In this context, it is essential to consider additional environmental variables, such as nutrient availability, in conjunction with salinity, which may influence the quantitative and qualitative composition of brown algae. The impact of salinity in conjunction with other abiotic factors on the metabolism of brown algae is reviewed in Section 3 [9,39].

### 2.6. Seasonality

Seasonality refers to the cyclic and predictable fluctuations in the environment that can impose pressure on organisms, compelling them to adapt accordingly [61]. Eleven articles were identified that explored seasonality as a potential parameter influencing the chemical composition of brown seaweeds (Table 6). Among the studied families, Sargassaceae (55%), Dictyotaceae (36%), and Scytosiphonaceae (27%) were significant. In terms of the monitored metabolites, phenolic compounds were the most prevalent (82%). The seasonal periods analyzed in the literature covered all the seasons: spring, summer, fall, and winter.

Gager et al. [62] analyzed the phenolic content of several species of algae—*A. esculenta*, *A. nodosum, F. serratus*, *H. elongata*, *L. ochroleuca, H. siliquosa*, and *B. bifurcata*. This study assessed temporal and seasonal variations and also explored the bioactivities associated with these species. Of all the species studied, the *Fucales* group (comprising *A. nodosum, H. elongata, F. serratus*, and *H. siliquosa*) exhibited the highest total phenolic content, up to three times greater than that of the *Laminariales* group (*A. esculenta* and *L. ochroleuca*). *A. nodosum* demonstrated the highest total phenolic content amongst all the algae, averaging 917 mg.g^−1^. Moreover, the study observed seasonal variations in phenolic content across these species except for *F. serratus* and *H. siliquosa*. Autumn typically showed the highest phenolic levels for most species of algae.

The antioxidant activity of *S. latissima* and its correlation with seasonality were investigated by Marinho et al. [65]. The contents of total phenolics and fucoxanthin were found to be higher during the autumn and winter months, while total flavonoids were more abundant in samples collected during the summer. The maximum concentrations of total phenolics, total flavonoids, and fucoxanthin were 2.41 mg GAE g^−1^ (in November), 4.56 mg RE g^−1^ DM (in September), and 665 μg g^−1^ DM (in January), respectively.

Cagalj et al. [66] analyzed the effect of seasonal growth (during the summer of 2020: May to September) on the antimicrobial and antioxidant activities of *C. compressa*. They noted that changes in total phenolic content, total tannin content, and antioxidant activity (measured using ferric reducing power or FRAP) correspond to variations throughout the growing season, reaching a peak in June when the algae were fully developed and featured highly branched thalli with aerocysts. The algae harvested in July and August demonstrated increased antimicrobial activity. Nonetheless, the highest recorded levels of phenolics (83.4 ± 4.0 mg GAE g^−1^) and total tannins (8.8 ± 0.8 mg CE g^−1^) were observed in June (during early summer). Moreover, the authors identified several compounds, with oleic acid (C18:1n-9) being the most abundant compound (over 15%) in all samples.

A study conducted by Cagalj et al. [64] explored the impact of seasonal variations on the total phenolic content and antioxidant activity of *P. pavonica.* They discovered that the maximum total phenolic content was observed in early summer, specifically in June (26.69 ± 1.86 mg GAE·g^−1^), contrasting to the lesser levels recorded in May, during spring. The predominant compounds during these periods were fatty acids, namely oleic acid, palmitic acid, and palmitoleic acid. The antioxidant activity likewise peaked in June; yet, it demonstrated decreased values by August.

Manino et al. [63] explored the correlation between ontogenetic stages, thallus morphology, and phenolic content throughout seasonal variations in *C. amentacea* and *D. polypodioides* collected off Sicily’s coast. The researchers discovered that life cycle stages and thallus morphology affect the total phenolic content of algae, observing higher phenolic levels when the algal thalli were in the adult stage. Specifically, *D. polypodioides* demonstrated the highest phenolic content during the winter and autumn (0.98% DW), while *C. amentacea* displayed this during the summer and spring (0.6% DW).

Mansur et al. [67] studied the impact of extraction method and seasonality on the metabolites of *C. tamariscifolia* and its cytotoxic activity against three cancer cell lines. They found that 100% methanol extracts were generally more effective in drawing out metabolites. Among these, polyphenols and flavonoids were the most plentiful, while proteins were the least. These findings were attributed to the polarity of secondary metabolites and the chosen extraction method. As for seasonal variations, phenolic levels were mostly higher in spring and summer, reaching 102.23 ± 1.85 mg.g^−1^ DW), with the lowest levels noted in autumn. The flavonoid content was the richest during spring, measuring 49.21 ± 4.83 mg.g^−1^ DW. The authors asserted that the algae’s chemical composition shift throughout the seasons is a response to increased herbivory and solar radiation in summer, leading to heightened metabolite content.

Seasonality is a cyclical and multifaceted abiotic parameter influenced by various abiotic environmental factors, such as seawater temperature, salinity, and nutrient availability. These factors often interact with each other [4]. Moreover, seasonality varies between different locations due to this broad range of intrinsic abiotic factors. This variation presents several methodological and research challenges for those seeking to derive ecological and environmental conclusions. Nonetheless, despite this complexity, some authors observed that the season with the highest phenolic production of secondary metabolites in brown algae tends to be consistent across various locations. In this context, algae from the genera *Alaria*, *Himanthalia*, *Laminaria*, *Bifurcaria*, *Halidrys*, *Saccharina*, and *Dictyopteris* exhibited the highest phenolic content in autumn [62,63,65]. In contrast, algae from the genus *Cystoseira* demonstrated higher levels of phenolics and tannins during the summer, as did algae from the genus *Padina* [63,64,66]. Additionally, studies monitoring flavonoids have observed their highest concentrations during the summer and spring [65,67]. Lastly, four other articles investigated seasonality as a factor impacting the chemical composition of brown algae, along with other abiotic factors [39,40,41,42]. These studies are reviewed in the subsequent section.

## 3. Interaction Between Multiple Abiotic Factors

Interactions between environmental variables in the marine environment are more the rule than the exception. Intense light intensifies heating in algae exposed at low tide and regulates carbon- and nitrogen-binding enzymes. Both temperature and salinity affect the mixing of deep and surface waters, thereby influencing nutrients and plankton migration. Furthermore, aspects like the carbonate system and ammonium availability are contingent on pH, salinity, and temperature. Cumulative effects, such as low salinity coupled with high temperature, can significantly impact the metabolic systems and chemical profiles of marine algae [4]. This paper includes studies that have examined the interaction between abiotic factors and their impact on the chemical composition of brown seaweed. Among these, the families Sargassaceae (40%), Dictyotaceae (30%), and Scytosiphonaceae (30%) were the most studied, with phenolic compounds being the most frequently analyzed (60%).

Kirk et al. [41] assessed the effects of temperature, light and irradiance, nutrients, and seasonality on the level of low-molecular-weight phlorotannins in fractions of *P. canaliculata*, *A. nodosum*, *F. vesiculosus*, and *H. elongata*. Their results showed that although the phenolic content peaks in the summer, local conditions have a more substantial influence than seasonal factors such as temperature and irradiance. Within this context, Mace Head was identified as the location presenting the highest phenolic concentrations for *P. canaliculata* (422.5 ± 23.6 μg PGE mg⁻^1^ DW), *A. nodosum* (312.2 ± 10.4 μg PGE mg⁻^1^ DW), and *H. elongata* (449.5 ± 12.6 μg PGE mg⁻^1^ DW). In contrast, *F. vesiculosus* exhibited the highest phenolic concentration at Finavarra (Ireland) (474.1 ± 3.3 μg PGE mg⁻^1^ DW). Alternatively, exposure to UVB radiation coupled with low PAR levels led to elevated phlorotannin concentrations (510 ± 27.4 μg PGE mg⁻^1^ DW). Moreover, treatments of 100 μM and 1 mM nitrogen in *F. vesiculosus* also increased these concentrations (450 μg PGE mg⁻^1^ DWE). These findings highlight the importance of UVB radiation and nitrogen availability in phlorotannin regulation, suggesting that optimizing these conditions may boost the production of bioactive compounds in algal cultures.

Endo et al. [36] studied how various factors influenced the color, pigment content, and sporophyte ratio of *U. pinnatifida*. They found that the highest pigment concentrations were observed under conditions of higher temperatures (15 °C), reduced light intensity (30 μmol photons m⁻^2^ s⁻^1^), and nutrient enrichment. Additionally, Endo et al. [43] explored the impact of these factors on *S. patens*, observing that while temperature directly influenced growth rates, it indirectly impacted phlorotannin concentrations. Interestingly, nutrient availability appeared to have an inverse relationship with the effects of temperature on phlorotannin levels and growth rate. Most notably, the condition with no nutrient supplementation at the lowest temperature (10 °C) resulted in the highest phlorotannin concentration, coming in at 4.5% of the DW.

Xu et al. [9] analyzed the influence of hydrodynamics, temperature, salinity, and nutrients on the phenolic compounds and bioactivity of various seaweeds, such as *P. arborescens, P. coriaceum, S. hemiphyllum, S. vachellianum, C. sinuosa*, and *E. binghamiae*. The study noted that unfavorable conditions, such as low salinity, limited dissolved oxygen, and nutrient-rich environments boosted antioxidant activity and phenolic content in brown algae. Meanwhile, tidal levels had a negligible effect. Significantly, *P. arborescens* displayed the highest total phenolic content (102.6 ± 3.6 GAE g⁻^1^) and antioxidant activity.

Yousefi et al. [39] examined the impact of salinity, seawater temperature, and seasonal variations on the fucoxanthin content and bioactivities of *D. indica, P. tenuis, I. stellata*, and *C. sinuosa*. These observations were made by comparing algae extracts collected during summer and winter. The authors concluded that in the summer, the fucoxanthin content and antioxidant activity were lower in extracts from all four species. This could be attributed to higher seawater temperatures and prolonged exposure to sunlight. In contrast, winter conditions, which featured the lowest monitored temperature and salinity, corresponded with higher fucoxanthin levels in *D. indica* (462.79 μg.g^−1^) and *I. stellata* (55.39 μg.g^−1^). Although no significant seasonal variations in antibacterial activity were observed, differences among species were noted, which the authors suggest is likely influenced by variations in fucoxanthin content.

De Jong et al. [49] examined the influence of light and nutrient availability on the chemical profile of *S. latissima*. They discovered that the highest sterol concentration was found in low-nutrient groups subjected to high light (502.4 ± 3.1 μmol photons m⁻^2^ s⁻^1^). The reaction to light was bidirectional. Certain compounds flourished under high light, while others favored low light conditions, depending on nutrient levels. Significantly, fucoxanthin and total sterols were more concentrated in algae with low nutrient enrichment when exposed to high light, yielding 3000 mg kg⁻^1^ of total sterols and 2500 mg kg⁻^1^ of fucoxanthin. Jevne et al. [50] identified no significant growth differences in *S. latissima* under varying natural light intensities (low and high); however, the highest protein concentration (77.0 ± 2.7 mg g⁻^1^ DW) was observed in algae subjected to low light levels (0–50 μmol m⁻^2^ s⁻^1^) and high-nutrient conditions.

Farasat et al. [40] studied the effect of temperature and seasonal variations on the antioxidant capacity of green, red, and brown algae found along the northern coast of the Persian Gulf. They found that brown algae showed the highest phenolic content and antioxidant activity in October, which coincided with increased seawater temperatures, pH, dissolved oxygen, and total nitrogen levels. Among all, *S. aqualifolium* had the maximum total phenolic concentration, almost 500 mg·100 g^−1^ DW, noted at a temperature of 30.534 °C ± 0.090. In contrast, *P. australis* showed a flavonoid content of 1000 mg·100 g^−1^ DW at a temperature of 13.967 °C ± 0.751. The study concluded that factors such as water temperature, dissolved oxygen, electrical conductivity, and pH have the most significant effect on antioxidant capacity.

Manino et al. [42] investigated the impact of temperature and seasonal variations on the chemical composition of brown algae, i.e., specifical fractions of *C. amentacea*, to evaluate the ongoing continuing effects of global warming on their habitats. They discovered that the total phenolic content (TPC) was higher in both summer and winter, with average values of 0.8 ± 0.07% and 0.76 ± 0.14% of DW, respectively. In laboratory experiments, *C. amentacea* exposed to a temperature of 25 °C showed a 39% increase in TPC after 8 h, while those at 30 °C noted only a 2.6% increase. The authors associated the rise in TPC with the activation and relocation of insoluble phlorotannins, improving the chemical defense of algae against elevated temperatures.

Lastly, Poza and colleagues [37] assessed the ideal abiotic conditions (such as temperature and nutrient availability) for the growth and biochemical characteristics of *L. marina*. The water extract had the highest phenolic concentration (0.99 ± 0.04 mg GAE/g), with chlorophyll A being the primary pigment. Both sporophytes and gametophytes displayed quicker growth at 1 PES and 10 PES concentrations; however, there was a distinct difference concerning the optimal temperature. Sporophytes flourished at 24 °C, while gametophytes exhibited superior growth at cooler temperatures (8 °C and 16 °C). Poza et al. determined that sporophytes demonstrate greater resilience to unfavorable nutrient conditions compared to gametophytes.

The literature suggests that to grasp how abiotic parameters impact the chemical composition of brown algae, one must take into account these factors both independently and collectively, as they mutually affect each other and shape the metabolic responses of the algae. In this context, seawater temperature is directly influenced by irradiance; enhanced radiation raises temperature [4]. Moreover, hydrodynamics, salinity, and light significantly contribute to nutrient availability [4,31,36,43,58]. These parameters can vary both temporally and spatially across various scales, from global and latitudinal to intermediate and local, including different oceanic zones [4]. Research shows that these abiotic elements not only affect the production of primary and secondary metabolites but also the growth and reproduction of macroalgae. The myriad responses of algae to environmental factors, which influence each other, complicate the creation of a model with predefined optimal conditions for growing brown algae to produce specific compounds.

## 4. Utilization of Abiotic Parameters for Bioprospecting of High-Value Bioactive Compounds

**Figure 2:**
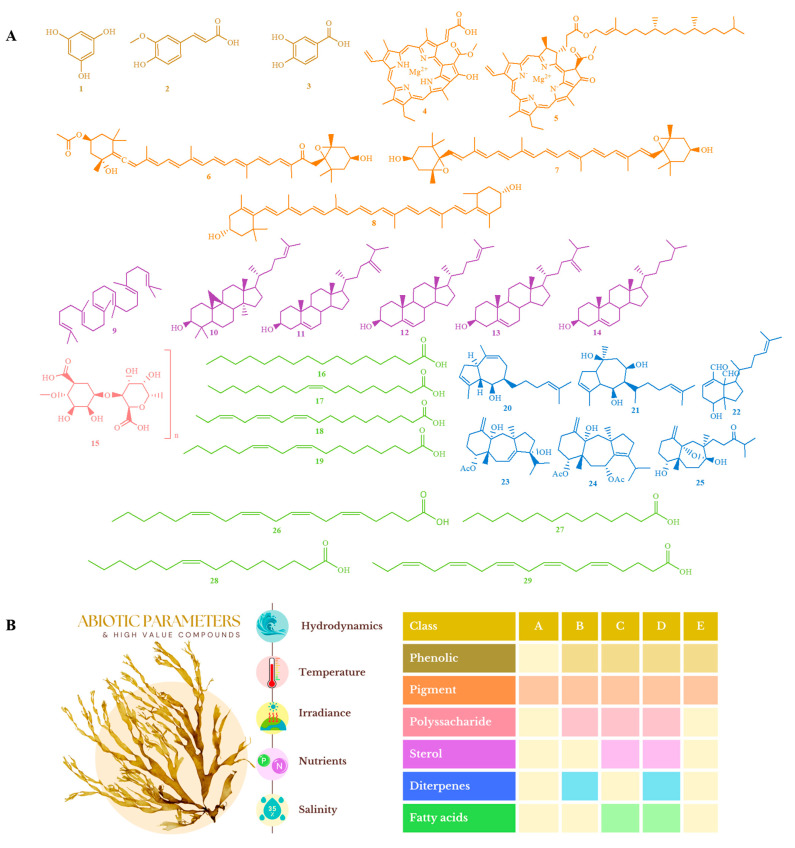
(**A**) High-value bioactive compounds identified under the influence of abiotic parameters: phloroglucinol (**1**), trans-ferulic acid (**2**), protocatechuic acid (**3**), chlorophyll c (**4**), chlorophyll a (**5**), fucoxanthin (**6**), violaxanthin (**7**), zeaxanthin (**8**), squalene (**9**), cycloartenol (**10**), fucosterol (**11**), 24-methylenecholesterol (**12**), desmosterol (**13**), cholesterol (**14**), alginate (**15**), palmitic acid (C16:0) (**16**), oleic acid (C18:1) (**17**), eicosatrienoic acid (C20:3n3) (**18**), linoleic acid (C18:2n6) (**19**), isopachydictyol a (**20**) and dictyol c (**21**), 5-hydroxy-1,6-cycloxenia-2,13-diene-16,17-dial (**22**), 4-acetoxy-9,14-dihydroxy-1,9-dolastane-1,9-diene (**23**), 4-acetoxy-14-hydroxy-dolastane-1(15),7,9-triene (**24**) and isolinearol (**25**), arachidonic acid (C20:4ω6) (**26**), myristic acid (C14:0) (**27**), palmitoleic acid (C16:1ω7) (**28**), and eicosapentaenoic acid (EPA) (**29**). (**B**) Graphic scheme assessed parameters in review articles, showing influences on metabolite classes: fatty acids (green), diterpenes (blue), sterols (purple), polysaccharides (pink), pigments (orange), and phenolic compounds (brown). Heatmap key: A (hydrodynamics), B (temperature), C (irradiance or light), D (nutrients), and E (salinity).

Changes in abiotic factors and their impact on the metabolism of brown algae have implications beyond ecological ones. Studying and understanding how these variables influence the metabolism of these algae is of significant relevance, particularly from a biotechnological perspective. Modifying these parameters in both natural cultures and controlled laboratory conditions can lead to opportunities for optimizing the production of metabolites of interest, resulting in increased yields of bioactive compounds. This is beneficial for bioprospecting processes, enhancing the efficient exploration of compounds while simplifying the maintenance of these species in the laboratory, thereby fostering their domestication. In this section, we highlight studies that identified and quantified compounds in relation to various parameters under consideration. The objective is to detail the chemical approaches in the studies more effectively, thereby stressing their significance in biotechnological applications (Figure 2).

The influence of temperature on the biosynthesis of phenolic compounds in brown algae is significant, as shown in several studies [38,40,42]. An increase in temperature enhances the production of these compounds, particularly under conditions of thermal stress, such as sudden temperature fluctuations. The exposure of these algae to diverse thermal gradients yields unique profiles of phenolic metabolites, emphasizing the need for optimization of cultivation conditions. Furthermore, this is key to exploring their potential biotechnological applications [68]. Generally referred to as polyphenols, these compounds are distinguished by one or more hydroxyl groups bearing aromatic rings. They have attracted significant attention due to their demonstrated antioxidant, antimicrobial, antidiabetic, anti-inflammatory, and anticancer properties in both in vitro and in vivo studies [69]. Polyphenols are classified into subclasses, including phenolic acids, flavonoids, stilbenes, and lignans, based on their chemical structures [70,71].

These compounds have attracted significant interest from both the scientific community and various industries, including pharmaceuticals, nutraceuticals, cosmetics, and food, thanks to their extensive bioactive potential. This interest brings about new opportunities for creating products aimed at improving human health [68]. Temperature substantially influences the production of these bioactive metabolites in marine algae. It is important to note that extreme conditions can result in decreased production. Therefore, it is essential to optimize this parameter in cultivation conditions to maximize biotechnological yields.

Mekinić et al. [38] explored the biological potential of the algae *D. dichotoma* and *P. pavonica*, as well as the influence of extraction methods—including the ultrasound technique and the type of solvent, relative to environmental temperature—on the phloroglucinol (1) content and phenolic acids. They also assessed their antioxidant activities. The extracts of *D. dichotoma* had a significantly higher content of phloroglucinol (1), with concentrations nearly triple in aqueous extracts as compared to the ethanolic ones. Conversely, the ethanolic extracts of *P. pavonica* contained much lower amounts, and it was impossible to quantify the compound in the aqueous extract due to its low levels. High-performance liquid chromatography (HPLC) analyses found that trans-ferulic acid (2) was the dominant phenol in *D. dichotoma*, while protocatechuic acid (3) predominated in *P. pavonica.* Extracts from *D. dichotoma* contained a greater quantify of hydroxycinnamic acid derivatives, while *P. pavonica* boosted a higher content of hydroxybenzoic acids.

The antioxidant activity was assessed through three assays: FRAP, DPPH, and ORAC. The results revealed that the ethanolic extracts of *D. dichotoma* exhibited the highest reducing activities, ranging between 690 to 792 mM TE. The antioxidant activities in other extracts were up to three times lower. The experimental data also indicate the presence of flavonoids in algae; concentrations reached up to 200 mg of quercetin equivalents per liter of extract (QE.L^−1^) in the aqueous extract of *P. pavonica* and ranged between 800–1000 mg QE.L^−1^ in the ethanolic extract of *D. dichotoma* at 20 °C. Farasat et al. 2023’s research [40] further supports the significant influence of temperature on flavonoid biosynthesis, showing that *Padina australis* exhibited concentrations reaching 1000 mg·100 g^−1^ DW. *Sargassum tenerrimum* demonstrated flavonoid levels of 800 mg·100 g^−1^ DW, while *S. aquifolium* and *Polycladia myrica* recorded concentrations of 1000 mg·100 g^−1^ DW and 600 mg·100 g^−1^ DW, respectively.

Irradiance is a parameter that profoundly influences the physiological aspects of marine macroalgae, affecting photosynthetic performance and chemical characteristics such as pigment concentration and lipid profile, which are tied to their developmental stages [72]. Proper management of irradiance in algal cultivation can augment pigment production, enhance the overall algae quality, and facilitate the bioprospecting of industrially relevant pigments [73]. More specifically, the relationship between irradiance, photosynthesis, pigment profile balance, environmental adaptation, and stress responses is particularly significant. A critical study by Endo et al. [36] further clarified these dynamics, offering priceless insights into the effects of irradiance on pigment synthesis and function.

The authors analyzed the irradiance involved in the cultivation of *U. pinnatifida*, quantifying the pigments using HPLC. They observed that an irradiance of 180 μmol photons m⁻^2^ s⁻^1^ resulted in reducing the Chl c (4)/Chl a (5) ratio, suggesting a photoprotective adaptation via the synthesis of pigments such as fucoxanthin (6), violaxanthin (7), and zeaxanthin (8). Among these pigments, fucoxanthin (6) plays a critical role in algae for absorbing light energy for photosynthesis while protecting chlorophyll from excessive light-exposure damage. This pigment has notably garnered attention for its health benefits, especially given its growing commercial viability in the natural products market and supplement segments [74]. To maximize these pigments’ production, managing cultivation conditions serves as a promising approach for algal biotechnology. Yet, it is crucial to prevent irradiance levels leading to photoinhibition by optimizing irradiance and other aspects, including temperature and nutrients, to maximize pigment output without endangering the photosynthetic viability of the algae [75].

The influence of irradiance on the production of steroid compounds in *Saccharina latissima* was notably documented by De Jong et al. [49]. Concentrations of squalene (9) and cycloartenol (10) significantly increased under high-irradiance conditions, independent of nutrient availability. Fucosterol (11) concentrations rose under high irradiance in low-nutrient groups but decreased in high-nutrient conditions. Similarly, the concentration of 24-methylenecholesterol (12) increased in low-nutrient settings under high irradiance while remaining unchanged in high-nutrient groups. In contrast, the desmosterol (13) concentration did not vary with high irradiance; its levels were significantly higher in low-nutrient conditions under high-light stress. Lastly, the concentration of cholesterol (14) remained unchanged under varying light conditions in both high- and low-irradiance environments. This suggests that, unlike other steroid compounds, the production of cholesterol (14) is not influenced by light stress.

The presence of phytosterols in seaweeds, specifically cholesterol (14), campesterol, fucosterol (11), β-sitosterol, and stigmasterol, may play an essential role in numerous beneficial biological functions. These sterols have demonstrated a bioactive role with confirmed potential to act as multi-targeted drugs, offering anticancer, antimicrobial, anti-inflammatory, and antioxidant effects [76]. Researchers have investigated these compounds for their ability to enhance the efficacy of steroid drug molecules, prevent drug resistance, and reduce the side effects that are often linked to pharmacological treatments. Future studies should aim to boost the bioavailability of these phytosterols in seaweeds, either in natural or in vitro settings, by adjusting different physicochemical parameters. This could pave the way for sustainable production of these metabolites [77].

Öztaşkent et al. [45] evaluated and concluded that irradiance significantly influences the composition of polysaccharides and fatty acids in algae. On one hand, blue light augments the absorption of inorganic carbon and boosts the production of alginate (15), a naturally occurring polysaccharide with beneficial health properties. This substance is extensively utilized in the nutraceutical and pharmaceutical sectors [78]. Notably, advancements in optimizing environmental conditions that increase alginate yield under the open sea or in vitro culture may be of substantial value to producers commercializing this product. In regards to fatty acids, palmitic acid (C16:0) (16) is present in high concentrations under all experimental conditions, and its synthesis is affected by irradiance. Oleic acid (C18:1) (17), the primary MUFA found in algae, has its production enhanced under red light, while eicosatrienoic acid (C20:3n3) (18), the most plentiful PUFA, is also synthesized in response to irradiance. Moreover, light influences the n-6 and n-3 fatty acids ratio, impacting the production of linoleic acid (C18:2n6) (19) under varying conditions. On average, seaweeds yield between 0.61% and 4.15% of DW in lipids. Some species display even higher yields, thereby making them an excellent source of unsaturated fats, especially polyunsaturated fatty acids (PUFAs). These PUFAs can offer substantial health benefits, particularly when considering the ω-6/ω-3 ratio in functional foods and nutraceuticals. Consequently, there is an increasing valuation of research aimed at defining optimal abiotic parameters for the bioprospecting of these metabolites.

Nutrients play a critical role in modulating metabolic profiles in macroalgae, inducing the biosynthesis of essential metabolites such as amino acids and proteins. In addition, they enhance the production of secondary metabolites, including phenolic compounds and diterpenes. These metabolites have significant environmental implications and are vital for increasing growth rates, which is reflected in augmented biomass and consequent production of metabolites with established biological activities, which is becoming a central factor in bioprospecting efforts. Celis-Plá et al. [54] studied the ecophysiological and metabolic stress responses of the brown alga *Cystoseira tamariscifolia* to excessive copper and nutrient enrichment. They identified several phenolic compounds via UHPLC, including shikimic acid, phloroglucinol, quinic acid, gallic acid, benzoic acid, quercetin, and kaempferol. Significantly, shikimic acid and phloroglucinol showed notable differences (*p* < 0.05) under high-copper conditions combined with nutrient enrichment. Also, there was an elevated expression of genes related to phenylalanine metabolism, which is crucial for synthesizing these phenolic compounds. The study’s antioxidant capacity was also evaluated using the DPPH method, revealing an increase under high-copper conditions regardless of nutrient enrichment. This amplified antioxidant capacity was connected to the activation of the glutathione-ascorbate cycle, as stated in previous research [79].

Multivariate analyses using generalized linear models (GLMs) for algae in the Dictyotaceae family have shown that the presence of nitrate and ammonia in the culture medium over time can significantly impact the production of diterpenes monitored and determined with RMN-^1^H and GCMS [52]. This includes compounds such as isopachydictyol A (**20**) and dictyol c (**21**), along with fucosterol. These studies underline the critical role of pH in regulating these compounds, including 5-hydroxy-1,6-cycloxenia-2,13-diene-16,17-dial (**22**), establishing pH as a key environmental variable for diterpenoid modulation in vitro. Conversely, the macroalga *C. cervicornis* presented as a useful model for producing 4-acetoxy-9,14-dihydroxy-1,9-dolastane-1,9-diene (**23**), with phosphorus as the main contributing factor. Other diterpenes, such as pachydictyol a in *D. menstrualis* and 4-acetoxy-14-hydroxy-dolastane-1(15),7,9-triene (**24**) and isolinearol (**25**) in *C. cervicornis*, were also identified as time-modulated under nutrient influence. Extracts from these algae, enriched in diterpenes and other isolated molecules, have exhibited notable biological activities [16,18,80]. Understanding how abiotic factors influence the profiles of these diterpenes is vital in establishing clean mono-algal culture stocks and reproducible bioreactors [19]. This knowledge promotes the extraction of high-quality and sustainable bioactive compounds for biotechnological applications.

Conversely, in relation to nutrient influence, Martins et al. [53] conducted an extensive analysis of the fatty acids in *D. menstrualis*, demonstrating that the total content was elevated in treatments with NO_3_^−^. This stimulated the production of polyunsaturated fatty acids such as arachidonic acid (C20:4ω6) (**26**). Palmitic acid (C16:0) (**16**) merged as the primary saturated fatty acid across all treatments, whereas myristic acid (C14:0) (**27**) exhibited variation based on the presence of NO_3_^−^ and CO_2_. Among the monounsaturated fatty acids, palmitoleic acid (C16:1ω7) (**28**) showed greater concentration levels in treatments with NO_3_^−^ and without CO_2_, whereas oleic acid (C18:1ω9) (**17**) was more copious in aerated treatments. Interestingly, the ratio of omega-3 fatty acids surpassed that of omega-6 in the treatments with NO_3_^−^. This investigation highlighted that both total and polyunsaturated fatty acid content in *D. menstrualis* were amplified in treatments with NO_3_^−^. This is notable, as the fatty acid content in the *Dictyota* genus species is often reported as low. Therefore, providing a nitrate source might be a vital parameter to consider for enhancing the extraction of polyunsaturated fatty acids from such algae, targeting biotechnological applications.

The analyzed studies also identified and analyzed fatty acids in *Cystoseira compressa* using UPLC-PDA-ESI-QTOF, with special emphasis on seasonality [66]. The most dominant compound identified was oleic acid (C18:1n-9) (**17**), which illustrated a content exceeding 15% in all tested samples and peaking in May. The month’s other two predominant compounds were palmitic acid (C16:0) (**16**) and palmitoleic acid (C16:1n-7) (**28**). Aside from the commonly represented fatty acids, the highest concentration of eicosapentaenoic acid (EPA) (**29**), an omega-3 fatty acid, was observed in July.

The composition of these fatty acids may be directly connected to the antioxidant and antibacterial activities evaluated in this study, implying that this algae possesses promising potential for applications in the pharmaceutical and cosmetic industries. Furthermore, the exploration of natural repositories should be approached considering both scholarly studies and bioprospecting intents. It is crucial to consider data tied to seasonal variations and interactions among multiple abiotic factors, such as temperature, salinity, and nutrient availability, which may affect the production of bioactive compounds. This will contribute to a more in-depth understanding of ecological dynamics and promote the sustainable development of algae-derived products.

## 5. Materials and Methods

To evaluate the ecological perspective on the chemical profile and its fluctuations in Phaeophyceae, a literature review was performed using the Web of Science database. The search spanned the period from January 2014 to March 2024 and utilized the following combinations of terms: “Brown seaweed” AND (“metabolites” OR “natural products”) AND (“temperature”) OR (“nutrient levels”) OR (“light” OR “irradiance”) OR “salinity” OR (“hydrodynamics” OR “tides” OR “water flow”) OR (“seasonal variations” OR “seasonality” OR “seasonal effects”). Furthermore, a supplemental search was conducted on Google Scholar, applying the same descriptors and time filter, to pinpoint relevant articles addressing the parameters not found during the preliminary search. Only original articles published in English were considered, while duplicate records and non-original publications were disregarded. The selection procedure was based on a thorough reading of titles and abstracts, centering on studies that analyzed the influence of abiotic factors on the chemical profile of brown algae.

The studies were organized according to relevant abiotic factors, with detailed information on family, genus, species, study area (sampling location), country, metabolite classes, and types of chemical extracts used. This information was compiled into tables and summarized in graphical representations. Additionally, we emphasized the highest recorded metabolite levels relative to variations in the evaluated parameters. This serves as a reference to recognize crucial connections between the ecological importance of these secondary metabolites and their potential for biotechnological applications.

## 6. Conclusions

The abiotic factors examined in this study—hydrodynamics, temperature, light and irradiance, nutrients, salinity, and seasonality—significantly influence the chemical composition of brown algae. They affect metabolites such as phenolic compounds (phlorotannins), fatty acids, sterols, pigments, diterpenes, and polysaccharides and also impact growth rates. These elements interact with each other, varying at different scales (global, regional, and local), to modulate the metabolic responses of the species.

In this review, it was found that hydrodynamics and salinity have a smaller influence on the production of metabolites, having an impact on pigments (hydrodynamics) and phenolic compounds (hydrodynamics and salinity), while nutrient input influences all chemical classes (phenolics, pigments, polysaccharides, sterols, diterpenes, and fatty acids). This point of view influences the research of natural products from brown algae, which focuses on obtaining products according to abiotic conditions.

Understanding how these factors influence the responses of brown algae in situ is essential, as these organisms can serve as bioindicators, reflecting changes in their environment. Additionally, recreating and comprehending their interactions under laboratory conditions is equally crucial, as algae produce metabolites with notable bioactivities, which include antioxidant, anti-inflammatory, and anticancer properties. Thus, understanding abiotic conditions is key to the sustainable development of natural products derived from these metabolites. The findings and data presented in this literature review may not only contribute to the sustainable development of economically important algal species but also aid in environmental modeling and conservation of vital species in marine ecosystems.

While this review analyzes a substantial number of studies, there is still a noticeable absence of research that independently explores abiotic parameters in relation to metabolic profiles. There are also gaps in studies proposing robust experimental designs for evaluating the interactions of these data. This is largely due to the difficulties associated with executing in situ studies, replicating conditions, and manipulating abiotic variables in lab settings. Therefore, our study implores researchers to develop inquires focused on evaluating these parameters, as the results could provide invaluable ecological and biotechnological insights.

## Figures and Tables

**Table 1:** The influence of hydrodynamics in Phaeophyceae seaweed. In this table, a systematic organization of data from the analyzed articles is presented, documenting only the highest recorded concentrations of metabolites in the column “Representative Content”. Chl: chlorophyll; DW: dry weight; EtOH: ethanol; FW: fresh weight; GAE: gallic acid equivalent; H_2_O: water; MDA: malondialdehyde; NR: unregistered; PC: phenolic content; Ref: reference.

Families	Species	Study Area	Type of Hydrodynamics	Extract Type	Analyzed Metabolite	Representative Content	Ref
Dictyotaceae	*Canistrocarpus cerviconis*	Ponta de Pedras Beach (Goiana) and Jaguaribe Beach (Itamaracá Island) (Brazil)	Tide exposure	Aqueous extract	PC	46.72 ± 1.44 mg GAE·g^−1^	[28]
Dictyotaceae	*Lobophora variegata*	Ponta de Pedras Beach (Goiana) and Jaguaribe Beach (Itamaracá Island) (Brazil)	Tide exposure	Aqueous extract	PC	36.45 ± 1.09 mg GAE.g-^1^	[28]
Dictyotaceae	*Pachydictyon coriaceum*	Haimen Bay, Honghai Bay, Shen’ao Bay, Jinghai Bay, Chishi Bay, (China)	High, middle, and low tides	Organic extract (EtOH/Acetone 1:20)	PC	Honghai Bay (low-tide zone): 54.0 ± 2.2 GAE.g^−1^	[9]
Fucaceae	*Ascophyllum nodosum*	Viana do Castelo (Portugal)	Middle tide	NR	Pigments, hydrogen peroxide, malondialdehyde, thiols, and proline	Carotenoids: 0.27 mg g^−1^ FW; MDA: 29 nm g^−1^ FW-low; Proline: 0.15 mg g^−1^ FW-high	[29]
Fucaceae	*Fucus serratus*	Viana do Castelo (Portugal)	Low tide	NR	Pigments, hydrogen peroxide, malondialdehyde, thiols, and proline	Carotenoids: 0.45 mg g^−1^ FW; MDA: 35 nm g^−1^ FW-low; Thiols: 3.5 umol g^−1^ FW-low; Proline: 0.25 mg g^−1^ FW-low	[29]
Fucaceae	*Pelvetia canaliculata*	Srednii Island in the Keret Archipelago (Kandalaksha Bay, White Sea, Russia)	Tidal cycle (high water–ebb tide–low water–rising tide)	Hidrorganic extract (Acetone/H_2_O 7:3)	Pigments, PC (phlorotannins), carbohydrates, and fatty acids	Chl A: 0.8–0.9 mg g^−1^ DW; Chl C: 0.2 mg g^−1^ DW; Pheophytin A: 0.15 mg g^−1^ DW; Carotenoids: 0.64 mg g^−1^ DW; Intracelular phlorotannins: 7.4% DW; Cell-wall associated phlorotannins: 0.7% DW	[30]
Fucaceae	*Pelvetia canaliculata*	Viana do Castelo (Portugal)	Low and high tide	NR	Pigments, hydrogen peroxide, malondialdehyde, thiols, and proline	Carotenoids: 0.47 mg g^−1^ FW-low; MDA: 40 nm g^−1^ FW-high; Thiols: 3.1 umol g^−1^ FW-low; Proline: 0.19 mg g^−1^ FW-high	[29]

**Table 2:** The influence of temperature in Phaeophyceae seaweed. In this table, a systematic organization of data from the analyzed articles is presented, documenting only the highest recorded concentrations of metabolites in the column “Representative Content”. CE: catechin equivalent; CHCl_3_: chloroform; Chl: chlorophyll; DCM: dichloromethane; DMF: dimethylformamide; DMSO: dimethyl sulfoxide; DW: dry weight; EtOH: ethanol; GAE: gallic acid equivalent; H_2_O: water; Hex: hexane; MeOH: methanol; NR: unregistered; PC: phenolic content; PGE: phloroglucinol equivalents; Ref: reference; TPC: total phenolic content.

Families	Species	Study Area	Temperature	Extract Type	Analyzed Metabolite	Representative Content	Ref.
Alariaceae	*Undaria pinnatifida*	Along the coast near Hirota Bay (38°95′ N, 141°67′ E) in Iwate Prefecture (Japan)	15 and 5 °C	Organic extract (DMF)	Pigments: Chl c1, Chl a, Chl c2, fucoxanthin, and violaxanthin + zeaxanthin	Chl c1: 0.025 mg.g^−1^ (15 °C), 0.017 mg.g^−1^ (5 °C); Chl a: 0.506 mg.g^−1^ (15 °C), ~0.4 mg.g^−1^ (5 °C); Chl c2: 0.027 (15 °C), 0.020 (5 °C); Fucoxanthin: 0.162 ± 0.008 mg.g^−1^ (15 °C), ~0.150 mg.g^−1^ (5 °C); Violaxanthin+zeaxanthin: 0.062 ± 0.020 mg.g^−1^ (15 °C), ~0.050 mg.g^−1^ (5 °C)	[36]
Chordariaceae	*Leathesia marina*	East coast of Nuevo Gulf (Patagonia)	8 °C, 16 °C, and 24 °C	Organic extracts (acetone, DMSO, MeOH, and aqueous)	PC, polysaccharides, and pigments	0.99 ± 0.04 mg GAE·g−1 (aqueous extract); Chl a: 0.46 ± 0.013 μg.mL^−1^ (80% acetone solvent)	[37]
Dictyotaceae	*Dictyota dichotoma*	Coast of Ciovo Island (Central Dalmatia, Croatia)	20 °C, 40 °C, and 60 °C	Aqueous and EtOH extracts	TPC and tannins	TPC: 300–350 GAE.L^−1^ (ethanolic extract −20 °C); Tannins: 0.3–0.4 CE.L^−1^ (ethanolic extract −20 °C)	[38]
Dictyotaceae	*Dictyota indica*	Intertidal zone of Qeshm Island, Persian Gulf (Iran)	37.6 °C and 23.8 °C	Aqueous and organic extracts (MeOH, Hex, CHCl_3_)	Pigments	462.79 ± 3.08 μg.g^−1^ (23.8 °C)	[39]
Dictyotaceae	*Dictyota menstrualis*	Experiment 1: Enseada do Forno, in Armação de Búzios (State of Rio de Janeiro, Brazil)	23.2 ± 0.17 °C (2018) in Enseada do Forno and 21 ± 1 °C under controlled conditions	DCM extracts	Pachydictyol A and 5-acetoxy-1,6-cycloxenia-2,13-diene-16,17-dial	Pachydictyol A: 8.79% (23.2 ± 0.17 °C), 15.10% (21 ± 1 °C); 5-acetoxy-1,6-cycloxenia-2,13-diene-16,17-dial: 21.98% (23.2 ± 0.17 °C), 24.04% (21 ± 1 °C)	[19]
Dictyotaceae	*Pachydictyon coriaceum*	Haimen Bay, Honghai Bay, Shen’ao Bay, Jinghai Bay, Chishi Bay (China)	24.23–29.97 °C	Organic extract (EtOH/Acetone 1:20)	PC	54.0 ± 2.2 GAE.g^−1^ (Honghai Bay)	[9]
Dictyotaceae	*Padina arborescens*	Honghai Bay (China)	24.23–29.97 °C	Organic extract (EtOH/Acetone 1:20)	PC	102.6 ± 3.6 GAE·g^−1^ extract	[9]
Dictyotaceae	*Padina australis*	Heleylah (coast of the Persian Gulf)	13.967 °C ± 0.751	MeOH extracts	TPC	100 mg·100 g^−1^ DW	[40]
Dictyotaceae	*Padina pavonica*	Coast of Ciovo Island (Central Dalmatia, Croatia)	20 °C, 40 °C, and 60 °C	Aqueous and ethanolic extract	TPC and tannis	TPC: 400–450 GAEL.L^−1^ (ethanolic extract−40 °C); Tannins: 0.1–0.2 CE.L^−1^ (aqueous extract −20 °C and 40 °C)	[38]
Dictyotaceae	*Padina tenuis*	Intertidal zone of Qeshm Island, Persian Gulf (Iran)	37.6 °C and 23.8 °C	Aqueous and organic extracts (MeOH, Hex, and CHCl_3_)	Pigments	42.77 ± 0.95 μg·g^−1^ (23.8 °C)	[39]
Fucaceae	*Ascophyllum nodosum*	Flaggy Shore, Finavarra, County Clare; Mace Head, County Galway (Ireland)	6–16 °C	MeOH extracts	PC: phlorotannins	Mace Head: 312.2 ± 1 0.4 μg PGE mg^−1^ DW (15 °C)	[41]
Fucaceae	*Fucus vesiculosus*	Flaggy Shore, Finavarra, County Clare; Mace Head, County Galway (Ireland)	6–16°C	MeOH extracts	PC: phlorotannins	Finavarra: 474.1 ± 3.3 μg PGE mg^−1^ DW (10 °C)	[41]
Fucaceae	*Pelvetia canaliculata*	Flaggy Shore, Finavarra, County Clare; Mace Head, County Galway (Ireland)	6–16 °C	MeOH extracts	PC: phlorotannins	Mace Head: 422.5 ± 23.6 g PGE mg^−1^ DW (10 °C)	[41]
Himanthaliaceae	*Himanthalia elongata*	Flaggy Shore, Finavarra, County Clare; Mace Head, County Galway (Ireland)	6–16 °C	MeOH extracts	PC: phlorotannins	Mace Head: 449.5 ± 12.6 μg PGE mg^−1^ DW (14 °C)	[41]
Sargassaceae	*Cystoseira amentacea*	Marine-Protected Area Capo Gallo-Isola delle Femmine (Scily)	25 °C and 30 °C	EtOH extracts	PC	t0 (25 and 30 °C): 0.77 ± 0.15% of DW; t2 (25 °C): 1.07 ± 0.01% of DW; t3 (25 °C): 0.96 ± 0.14% of DW; t4 (25 °C): 0.44 ± 0.17% of DW	[42]
Sargassaceae	*Polycladia myrica*	Rostami (coast of the Persian Gulf)	30.534 °C ± 0.091	MeOH extracts	TPC	150 mg·100 g^−1^ DW	[40]
Sargassaceae	*Sargassum aquifolium*	Rostami (coast of the Persian Gulf)	30.534 °C ± 0.088	Methanolic extracts	TPC	500 mg·100 g^−1^ DW	[40]
Sargassaceae	*Sargassum hemiphyllum*	Shen’ao Bay (China)	24.23–29.97 °C	Organic extract (EtOH/Acetone 1:20)	PC	75.7 ± 1.0 GAE.g^−1^ extract	[9]
Sargassaceae	*Sargassum ilicifolium*	Rostami (coast of the Persian Gulf)	30.534 °C ± 0.088	Methanolic extracts	TPC	369.69 mg GAE·g^−1^	[40]
Sargassaceae	*Sargassum patens*	Sheltered site along the coast at Unosaki (39°51′20″ N, 139°48′56″ E), Akita Prefecture (Japan)	10 °C, 20 °C and 30°C	Hidroorganic extracts (MeOH:H_2_O, 8:2)	PC: phlorotannins	4.5% DW (10 °C)	[43]
Sargassaceae	*Sargassum tenerrimum*	Rostami (coast of the Persian Gulf)	30.534 °C ± 0.088	Methanolic extracts	TPC	308.810 ± 5.064 mg GAE·g^−1^	[40]
Sargassaceae	*Sargassum vachellianum*	Honghai Bay, Shen’ao Bay, Jinghai Bay, and Jiazi Bay (China)	24.23–29.97 °C	Organic extract (EtOH/Acetone 1:20)	PC	Honghai Bay: 61.9 ± 1.1 GAE.g^.−1^	[9]
Sargassaceae	*Sirophysalis trinodis*	Rostami (coast of the Persian Gulf)	30.534 °C ± 0.091	MeOH extracts	TPC	100 mg·100 g^−1^ DW	[40]
Scytosiphonaceae	*Colpomenia sinuosa*	Heleylah (coast of the Persian Gulf)	20.467 °C ± 0.328	MeOH extracts	TPC	100 mg·100 g^−1^ DW	[40]
Scytosiphonaceae	*Colpomenia sinuosa*	Shen’ao Bay (China)	24.23–29.97 °C	Organic extract (EtOH/Acetone 1:20)	PC	69.4 ± 1.7 GAE.g^−1^ extract	[9]
Scytosiphonaceae	*Colpomenia sinuosa*	Intertidal zone of Qeshm Island, Persian Gulf	37.6 °C and 23.8 °C	Aqueous and organic extracts (MeOH, Hex, and CHCl_3_)	Pigments	19.23 ± 0.0.65 μg.g^−1^ (23.8 °C)	[39]
Scytosiphonaceae	*Endarachne binghamiae*	Haimen Bay (China)	24.23–29.97 °C	Organic extract (EtOH/Acetone 1:20)	PC	36.5 ± 1.4 mg GAE·g^−1^ extract	[9]
Scytosiphonaceae	*Iyengaria stellata*	Intertidal zone of Qeshm Island, Persian Gulf	37.6 °C and 23.8 °C	Aqueous and organic extracts (MeOH, Hex, and CHCl_3_)	Pigments	55.39 ± 1.15 μg.g^−1^ (23.8 °C)	[39]

**Table 4:** The influence of nutrients in Phaeophyceae seaweed. In this table, a systematic organization of data from the analyzed articles is presented, documenting only the highest recorded concentrations of metabolites in the column “Representative Content”. Ae: aeration; CO_2_: carbon dioxide; CHCl_3_: chloroform; Chl: chlorophyll; CuSO_4_·5H_2_O: copper(II) sulfate pentahydrate; DCM: dichloromethane; DMF: dimethylformamide; DMSO: dimethyl sulfoxide; DW: dry weight; EE: enriched experiment; EtOH: ethanol; GAE: gallic acid equivalent; H_2_O: water; KH_2_PO_4_: dihydrogen potassium; KNO_3_: potassium nitrate; MeOH: methanol; N: nitrogen; NE: non-enriched experiment; NH_4_: ammonium ion; NO_3_^−^: nitrate ion; NR: unregistered; PC: phenolic content; PES: Provasoli-enriched seawater; PO_4_: phosphate ion; Ref: reference; RGR: relative to growth rate; VES: Von Stosch’s solution.

Families	Species	Study Area	Nutrients Concentration	Growth Rate	Extract Type	Analyzed Metabolite	Representative Content	Ref
Alariaceae	*Undaria pinnatifida*	Hirota Bay (Japan)	PES: 25%	−0.150–0.104 ± 0.657–0.739% day^−1^ (non-enriched)	Organic extract (DMF)	Pigments: Chl a, Chl c1; Chl c2; fucoxanthin, violacanthin + zeaxanthin	Chl a: 0.506 mg·g^−1^ (EE), ~0.3 (NE); Chl c1: 0.025 mg.g^−1^ (EE), ~0.01 mg·g^−1^ (NE); Chl c2: 0.027 mg·g^−1^ (EE), ~0.01 mg·g^−1^ (NE); Fucoxanthin: 0.162 mg.g^−1^ (EE), ~0.1 mg·g^−1^ (NE); Violaxanthin + zeaxanthin: 0.062 mg g^−1^ (EE), 0.04 mg·g^−1^ (NE)	[36]
Chordariaceae	*Leathesia marina*	Patagonian coast (Argentina)	PES: 0, 0.2, 2, and 20 mL PES/L	1.75 ± 0.15 (PES 20mL/L) to sporophyte and 0.99 ± 0.27 (PES 2 mL/L) to gametophyte	Organic extracts (acetone, DMSO, and MeOH) and aqueous extract	PC, polysaccharides and pigments	Carbohydrates: 19.9 ± 2.4 mg · 100 mg^−1^	[37]
Dictyotaceae	*Canistrocarpus cerviconis*	Angra dos Reis (Brazil)	PES/2 and sterilized seawater	1.39% day^−1^ (control culture—7 days) and 0.24% day^−1^ (enriched culture—7 days)	DCM extract	Diterpenes	4-acetoxy-9,14-dihydroxy-1,9-dolastane-1,9-diene: 29.573 ± 2.928 (EE after 7 days); Isolinearol: 10.424 ± 2.143 (EE after 14 days); 4-acetoxy-14-hydroxy-dolastane-1(15),7,9-triene: 3.821 ± 1.020 (EE after 7 days)	[52]
Dictyotaceae	*Dictyota menstrualis*	Angra dos Reis (Brazil)	PES/2 and sterilized seawater	NR	DCM extract	Diterpenes and sterols	Fucosterol: 40.12 ± 1.52% (EE after 56 days); Pachydictyol A: 38.307 ± 3.485 (control after 14 days)	[52]
Dictyotaceae	*Dictyota menstrualis*	Rio do Fogo (Rio Grande do Norte, Brazil)	VSES/2 with and without NO_3_^−^, CO_2_, injection and aeration	16.5% day^−1^ NO_3_^−^ + Ae)	Hidroorganic extract (CHCl_3_:MeOH:H_2_O 2:2:1)	Pigments, carbohydrates, lipids, and fatty acids	Chl a: 0.4 mg.g^−1^ DW (all treatments containing NO_3_^−^); Carbohydrates: 460 mg·g^−1^ (N + CO_2_); Lipids: 175 mg·g^−1^ (N + CO_2_); Fatty acids: 18.86 ± 1.60 mg·g^−1^ (N + Ae)	[53]
Dictyotaceae	*Pachydictyon coriaceum*	Haimen Bay, Honghai Bay, Shen’ao Bay, Jinghai Bay, Chishi Bay (China)	Ammonia (0.01–0.20 mg/L), nitrate (0.04–0.47 mg/L), nitrogen (0.00 –0.06 mg/L), and phosphate (0.02–0.08 mg/L)	NR	Organic extract (EtOH/Acetone 1:20)	PC	Honghai Bay: 54.0 ± 2.2 GAE·g^−1^	[9]
Dictyotaceae	*Padina arborescens*	Honghai Bay (China)	Ammonia (0.01–0.20 mg/L), nitrate (0.04–0.47 mg/L), nitrogen (0.00 –0.06 mg/L), and phosphate (0.02–0.08 mg/L)	NR	Organic extract (EtOH/Acetone 1:20)	PC	102.6 ± 3.6 GAE·g^−1^ extract	[9]
Laminariaceae	*Saccharina latissima*	Sea farm (Norway)	High and Low	0.23 ± 0.03 day^−1^ (low nutrients) to 0.66 ± 0.06 mm day^−1^ (high nutrients)	NR	Protein	77.0 ± 2.7 mg·g^−1^ DW	[50]
Laminariaceae	*Saccharina latissima*	Commercial cultivator Zeewaar (Netherlands)	Amonio and phosphate: 51 μM NH_4_, 148 μM NO_3_, and 25 μM PO_4_	NR	DCM extract	Sterols	Total Sterols: 2.443 ± 227 mg·kg^−1^; Fucosterol: 2.000 mg·kg^−1^	[49]
Sargassaceae	*Cystoseira tamariscifolia*	United Kingdom (50°36′N, 4°42′W)	CuSO_4_·5H_2_O at 0.5 μM (low copper levels) and 2.0 μM (high copper levels), and nutrient conditions at control or natural seawater, and at 50 μM KNO_3_ + 5 μM KH_2_PO_4_	NR	Hydromethanolic (MeOH:H_2_O, 8:2)	Pigments, carbohydrates, lipids, and fatty acids	Chl a: 2.6 mg·g^−1^; Chl c: 0.4 mg·g^−1^	[54]

**Table 5:** The influence of salinity in Phaeophyceae seaweed. In this table, a systematic organization of data from the analyzed articles is presented, documenting only the highest recorded concentrations of metabolites in the column “Representative Content”. CHCl_3_: chloroform; DM: dry matter; DW: dry weight; EtOH: ethanol; GAE: gallic acid equivalent; Hex: hexane; MeOH: methanol; NR: unregistered; ppt: parts per trillion; Ref: reference; TPC: total phenolic content.

Families	Species	Study Area	Evaluated Salinity	Extract Type	Analyzed Metabolite	Representative Content	Ref
Dictyotaceae	*Dictyota australis*	Thevenard Island (Australia)	35.15	MeOH extract	TPC	1.63 ± 0.34% DM	[56]
Dictyotaceae	*Dictyota ceylanica*	Thevenard Island (TI) and Exmouth Gulf (EG) (Australia)	35.15 (TI) and 35.05 (EG)	MeOH extract	TPC	0.35 ± 0.03% DM	[56]
Dictyotaceae	*Dictyota ciliolata*	Port Gregory (Australia)	35.66	MeOH extract	TPC	0.95 ± 0.45% DM	[56]
Dictyotaceae	*Dictyota indica*	Intertidal zone of Qeshm Island, Persian Gulf (Iran)	37.84 and 30.62 ppt	MeOH: Hex, H_2_O, and CHCl_3_ extracts	Fucoxanthin	30.62 ppt: 462.79 ± 3.08 μg g^−1^; 37.84 ppt: 210.72 ± 1.20 μg g^−1^	[39]
Dictyotaceae	*Dictyopteris muelleri*	Jurien Bay (Australia)	35.66	MeOH extract	TPC	0.48 ± 0.03% DM	[56]
Dicyotaceae	*Dictyota naevosa*	Port Gregory (Australia)	35.66	MeOH extract	TPC	1.06 ± 0.43% DM	[56]
Dictyotaceae	*Dictyota sp.*	Port Gregory (Australia)	35.66	MeOH extract	TPC	0.43 ± 0.03% DM	[56]
Dictyotaceae	*Dilophus sp.*	Port Gregory (Australia)	35.66	MeOH extract	TPC	1.11 ± 0.14% DM	[56]
Dictyotaceae	*Distrominium sp.*	Port Gregory (PG) and Jurien Bay (JB) (Australia)	35.66	MeOH extract	TPC	PG: 2.24 ± 0.38% DM; JB: 4.40 ± 0.72% DM	[56]
Dictyotaceae	*Lobophora variegata*	Eagle Bay (EaG) and Exmouth Gulf (ExG) (Australia)	EaB: 35.76 and ExG: 35.05	MeOH extract	TPC	ExG: 0.31 ± 0.01% DM; EaB: 8.73 ± 1.05	[56]
Dictyotaceae	*Lobophora variegata*	El Hierro Island (S1 and S2) and Gran Canaria (S3 for control) (Spain)	S1: 36.74 ± 0.11- 34.70 ± 0.10; S2: 36.75 ± 0.01–35.30 ± 0.08; S3 (control): 35.12 ± 0.30–35.08 ± 0.01	MeOH extract	TPC	S1: 40–90 mg.g^−1^ DW; S2: 30–140 mg.g^−1^ DW; S3: 50–90 mg.g^−1^ DW	[57]
Dictyotaceae	*Pachydictyon coriaceum*	Haimen Bay, Honghai Bay (HB), Shen’ao Bay, Jinghai Bay, Chishi Bay (China)	22.00–34.91	Organic extract (EtOH/Acetone 1:20)	TPC	HB: 54.0 ± 2.2 GAE.g^−1^	[9]
Dictyotaceae	*Padina arborescens*	Honghai Bay (China)	22.00–34.91	Organic extract (EtOH/Acetone 1:20)	TPC	102.6 ± 3.6 GAE/g extract	[9]
Dictyotaceae	*Padina pavonica*	El Hierro Island (S1 and S2) and Gran Canaria (S3 for control) (Spain)	S1: 36.74 ± 0.11–34.70 ± 0.10; S2: 36.75 ± 0.01–35.30 ± 0.08; S3 (control): 35.12 ± 0.30–35.08 ± 0.01	MeOH extract	TPC	S1: 10–20 mg.g^−1^ DW; S2: 5–10 mg.g^−1^ DW; S3: 5–20 mg.g^−1^ DW	[57]
Dictyotaceae	*Padina tenuis*	Intertidal zone of Qeshm Island, Persian Gulf (Iran)	37.84 and 30.62 ppt	MeOH: Hex, H_2_O, and CHCl_3_ extracts	Fucoxanthin	30.62 ppt: 42.77 ± 0.95 μg g^−1^; 37.84 ppt: 17.61 ± 0.43 μg g^−1^	[39]
Dictyotaceae	*Padina sp.*	Cygnet Bay (CB), Thevenard Island (TI) and Exmouth Gulf (ExG) (Australia)	CB: 35.07, TI: 35.15, and ExG: 35.05	MeOH extract	TPC	CB: 2.18 ± 0.17% DM; TI: 1.73 ± 0.03% DM; ExG: 1.95 ± 0.11% DM	[56]
Dictyotaceae	*Spatoglossum macrodontum*	Jurien Bay (Australia)	35.66	MeOH extract	TPC	1.81 ± 0.35% DM	[56]
Dictyotaceae	*Zonaria turneriana*	Jurien Bay (Australia)	35.66	MeOH extract	TPC	4.78 ± 0.39% DM	[56]
Lessionaceae	*Ecklonia radiata*	Jurien Bay (Australia)	25.66	MeOH extract	TPC	3.37 ± 0.28% DM	[56]
Sargassaceae	*Cystophora*	Eagle Bay (Australia)	35.76	MeOH extract	TPC	9.36 ± 0.68% DM	[56]
Sargassaceae	*Cystophora grevillei*	Eagle Bay (Australia)	35.76	MeOH extract	TPC	10.99 ± 2.63% DM	[56]
Sargassaceae	*Hormophysa cuneiformis*	Cygnet Bay (CB), Exmouth Gulf (ExG), Shark Bay (SB) (Australia)	CB: 35.07; ExG: 35.05; SB: 39–53.5	MeOH extract	TPC	CB: 2.59 ± 0.19% DM; ExG: 0.72 ± 0.10% DM; SB: 0.76 ± 0.12% DM	[56]
Sargassaceae	*Myriodesma serrulata*	Jurien Bay (Australia)	35.66	MeOH extracts	TPC	1.17 ± 0.15% DM	[56]
Sargassaceae	*Sargassopsis decurrens*	Thevenard Island (TI) and Shark Bay (SB) (Australia)	TI: 35.15 and SB: 39–53.5	MeOH extracts	TPC	TI: 0.51 ± 0.07% DM; SB: 0.99 ± 0.12% DM	[56]
Sargassaceae	*Sargassum hemiphyllum*	Shen’ao Bay (China)	22.00–34.91	Organic extract (EtOH/Acetone 1:20)	TPC	75.7 ± 1.0 GAE/g extract	[9]
Sargassaceae	*Sargassum ligulatum*	Cygnet Bay, Thevenard Island and Exmouth Gulf (Australia)	CB: 35.07, TI: 35.15, and ExG: 35.05	MeOH extract	TPC	CB: 1.13 ± 0.14% DM; TI: 0.25 ± 0.03% DM; ExG: 0.33 ± 0.04% DM	[56]
Sargassaceae	*Sargassum linearifolium*	Exmouth Gulf (Australia)	35.05	MeOH extract	TPC	4.16 ± 0.75% DM	[56]
Sargassaceae	*Sargassum marginatum*	Thevenard Island (Australia)	35.15	MeOH extract	TPC	0.70 ± 0.11% DM	[56]
Sargassaceae	*Sargassum oligocystum*	Port Gregory (Australia)	35.66	MeOH extract	TPC	0.28 ± 0.11% DM	[56]
Sargassaceae	*Sargassum paradoxum*	Port Gregory (PG) and Jurien Bay (JB) (Australia)	35.66	MeOH extract	TPC	PG: 1.28 ± 0.10% DM; JB: 2.94 ± 0.27% DM	[56]
Sargassaceae	*Sargassum polycystum*	Cygnet Bay (Australia)	35.07	MeOH extract	TPC	1.42 ± 0.14% DM	[56]
Sargassaceae	*Sargassum polyphyllum*	Exmouth Gulf (Australia)	35.05	MeOH extract	TPC	0.63 ± 0.08% DM	[56]
Sargassaceae	*Sargassum rasta*	Cygnet Bay (Australia)	35.07	MeOH extract	TPC	0.81 ± 0.05% DM	[56]
Sargassaceae	*Sargassum vachellianum*	Honghai Bay (HB), Shen’ao Bay, Jinghai Bay and Jiazi Bay (China)	22.00–34.91	Organic extract (EtOH/Acetone 1:20)	TPC	HB: 61.9 ± 1.1 GAE/g extract	[9]
Sargassaceae	*Scaberia agardhii*	Eagle Bay (Australia)	35.76	MeOH extract	TPC	4.73 ± 0.65% DM	[56]
Sargassaceae	*Sirophysalis trinodis*	Cygnet Bay (CB), Thevenard Island (TI), Exmouth Gulf (ExG), Shark Bay (SB) and Eagle Bay (EaB) (Australia)	CB: 35.07; TI: 35.15; ExG: 35.05; SB: 39–53.5; EaB: 35.76	MeOH extracts	TPC	CB: 1.14 ± 0.15% DM; TI: 0.87 ± 0.19% DM; ExG: 0.55 ± 0.12% DM; SB: 1.05 ± 0.34% DM; EaB: 3.56 ± 0.92% DM	[56]
Sargassaceae	*Turbinaria gracilis*	Cygnet Bay (Australia)	35.07	MeOH extracts	TPC	2.96 ± 0.28% DM	[56]
Scytosiphonaceae	*Colpomenia sinuosa*	Shen’ao Bay (China)	22.00–34.91	Organic extract (EtOH/Acetone 1:20)	TPC	69.4 ± 1.7 GAE/g extract	[9]
Scytosiphonaceae	*Colpomenia sinuosa*	Intertidal zone of Qeshm Island, Persian Gulf (Iran)	37.84 and 30.62 ppt	MeOH: Hex, H_2_O, and CHCl_3_ extracts	Fucoxanthin	30.62: 19.23 ± 0.0.65 μg g^−1^; 37.84: 13.53 ± 0.35 μg g^−1^	[39]
Scytosiphonaceae	*Endarachne binghamiae*	Haimen Bay (China)	22.00–34.91	Organic extract (EtOH/Acetone 1:20)	TPC	36.5 ± 1.4 mg GAE/g extract	[9]
Scytosiphonaceae	*Iyengaria stellata*	Intertidal zone of Qeshm Island, Persian Gulf (Iran)	37.84 and 30.62 ppt	MeOH: Hex, H_2_O, and CHCl_3_ extracts	Fucoxanthin	30.62: 55.39 ± 1.15 μg g^−1^; 37.84: 26.10 ± 0.85 μg g^−1^	[39]
Sporochnaceae	*Sporochnus moorei*	Shark Bay (Australia)	39–53.5	Methanolic extract	TPC	1.15 ± 0.32% DW	[56]

**Table 6:** The influence of seasonality in Phaeophyceae seaweed. In this table, a systematic organization of data from the analyzed articles is presented, documenting only the highest recorded concentrations of metabolites in the column “Representative Content”. CE: catechin equivalents; CHCl_3_: chloroform; DM: dry matter DW: dry weight; EtAc: ethyl acetate; EtOH: ethanol; GAE: gallic acid equivalent; H_2_O: water; NR: unregistered; PC: phenolic content; PGE: phloroglucinol equivalents Ref: reference; TFC: total flavonoids content; TPC: total phenolic content; TTC: total tannins content.

Families	Species	Study Area	Seasonality Period	Extract Type	Analyzed Metabolite	Representative Content	Ref
Alariaceae	*Alaria esculenta*	Coastline by SME C-WEED Aquaculture (France)	Winter, spring, summer, and fall 2015–2017	Hydroethanolic extract (EtOH:H_2_O 1:1)	PC	Fall: 313 mg·g^−1^ DW	[62]
Dictyotaceae	*Dictyopteris polypodioides*	Carini, Palermo (Italy)	Winter, spring, summer, and fall	EtOH extract	PC	Winter: 0.98% DW	[63]
Dictyotaceae	*Dictyota indica*	Intertidal zone of Qeshm Island, Persian Gulf (Iran)	Summer and winter 2016	MeOH extract	Pigments	Winter: 462.79 ± 3.08 µg g^−1^ DW; Summer: 210.72 ± 1.20 µg g^−1^ DW	[39]
Dictyotaceae	*Padina australis*	Heleylah (Iran)	Winter	MeOH extract	TPC and Flavonoids	TPC: 100 mg.100 g^−1^; Flavonoids: 100 mg.100 g^−1^	[40]
Dictyotaceae	*Padina pavonica*	Gotnji Dolac (Croatia)	Summer 2020	Hydroethanolic extract (EtOH:H_2_O 1:1)	TPC	June: 26.69 ± 1.86 mg GAE·g−1	[64]
Dictyotaceae	*Padina tenuis*	Intertidal zone of Qeshm Island, Persian Gulf (Iran)	Summer and winter 2016	MeOH extract	Pigments	Winter: 42.77 ± 0.95 µg g^−1^ DW; Summer:17.61 ± 0.43 µg g^−1^ DW	[39]
Fucaceae	*Ascophyllum nodosum*	Coastline by SME C-WEED Aquaculture (France)	Winter, spring, and fall 2015–2017	Hydroethanolic extract (EtOH:H20 1:1)	PC	Summer: >917 mg·g^−1^ DW	[62]
Fucaceae	*Ascophyllum nodosum*	Flaggy Shore, Finavarra, County Clare; Mace Head, County Galway (Ireland)	Samples were collected between fall 2014 and winter 2015	MeOH extract	PC: phlorotannins	Mace Head: 312.2 ± 1 0.4 μg PGE mg^−1^ DW	[41]
Fucaceae	*Fucus serratus*	Coastline by SME C-WEED Aquaculture (France)	Winter, spring, and fall 2015–2017	Hydroethanolic extract (EtOH:H20 1:1)	PC	Summer: 350mg·g^−1^ DW	[62]
Fucaceae	*Fucus vesiculosus*	Flaggy Shore, Finavarra, County Clare; Mace Head, County Galway (Ireland)	Samples were collected between fall 2014 and winter 2015	MeOH extract	PC: phlorotannins	Finavarra: 474.1 ± 3.3 μg PGE mg^−1^ DW	[41]
Fucaceae	*Pelvetia canaliculata*	Flaggy Shore, Finavarra, County Clare; Mace Head, County Galway (Ireland)	Samples were collected between fall 2014 and winter 2015	MeOH extract	PC: phlorotannins	Mace Head: 422.5 ± 23.6 g PGE mg^−1^ DW	[41]
Himanthaliaceae	*Himanthalia elongata*	Flaggy Shore, Finavarra, County Clare; Mace Head, County Galway (Ireland)	Samples were collected between fall 2014 and winter 2015	Methanolic extract	PC: phlorotannins	Mace Head: 449.5 ± 12.6 μg PGE mg^−1^ DW	[41]
Himanthaliaceae	*Himanthalia elongata*	Coastline by SME C-WEED Aquaculture (France)	Winter, spring, summer, and fall 2015–2017	Hydroethanolic extract (EtOH:H_2_O 1:1)	PC	Fall: >750 mg·g^−1^ DW	[62]
Laminariaceae	*Laminaria ochroleuca*	Coastline by SME C-WEED Aquaculture (France)	Winter, spring, summer, and fall 2015–2017	Hydroethanolic extract (EtOH:H_2_O 1:1)	PC	Fall: <100 mg·g^−1^	[62]
Laminariaceae	*Saccharina latissima*	The integrated multi-trophic aquaculture (Danish)	Samples were collected between summer 2013 and summer 2014	Organic extracts (MeOH and EtOAc)	TPC, TFC, and fuxocanthin	TPC: 2.41 mg GAE g^−1^ (November); TFC: 4.56 RE g^−1^ DM (September); Fucoxanthin: 665 μg g^−1^ DM (January)	[65]
Sargassaceae	*Bifurcaria bifurcata*	Coastline by SME C-WEED Aquaculture (France)	Winter, spring, summer, and fall 2015–2017	Hydroethanolic extract (EtOH 1:1)	PC	Fall: 350 mg·g^−1^ DW	[62]
Sargassaceae	*Cystoseira amentacea*	Marine-Protected Area Capo Gallo-Isola (Sicily)	Winter, spring, summer, and fall	EtOH extract	PC	0.8 ± 0.07 and 0.76 ± 0.14% of DW	[42]
Sargassaceae	*Cystoseira amentacea*	Carini, Palermo (Italy)	Winter, spring, and fall	EtOH extract	PC	Summer: 0.6% DW	[63]
Sargassaceae	*Cystoseira compressa*	Gornji Dolac (Croatia)	Summer 2020	Hydroethanolic extract (EtOH:H_2_O 1:1)	TPC and TTC	TPC: 83.4 ± 4.0 MG GAE/G; TTC: 8.8 ± 0.8 mg CE/G	[66]
Sargassaceae	*Cystoseira tamariscifolia*	Shallow subtidal at Hannafore Point, Cornwall (United Kingdom)	Summer (June); fall (mid-October); winter (March); spring (early May)	Organic (MeOH and CHCl_3_), hydromethanolic (MeOH:H_2_O 7:3), and aqueous extracts	TPC, TFC, and polysaccharides	TPC: 102.23 ± 1.85 mg g^−1^ DW (MeoH 100% extract—summer); TFC: 49.21 ± 4.83 mg g^−1^ DW (chloroform extract—spring); Polysaccharides: 48.84 ± 3.66 mg g^−1^ DW	[67]
Sargassaceae	*Halidrys siliquosa*	Coastline by SME C-WEED Aquaculture (France)	Winter, spring, summer, and fall 2015–2017	Hydroethanolic extract (EtOH:H_2_O 1:1)	PC	Fall: >841 mg·g^−1^ DW	[62]
Sargassaceae	*Polycladia myrica*	Rostami (Iran)	Fall (October) and winter (December)	MeOH extract	TPC and TFC	TPC: 110 mg·100 g^−1^; TFC: 580 mg·100g^−1^	[40]
Sargassaceae	*Sargassum aquifolium*	Rostami (Iran)	Fall (October) and winter (December)	MeOH extract	TPC and TFC	TPC: 502.430 ± 30.855 mg·100 g^−1^; TFC: 980 mg·100g^−1^	[40]
Sargassaceae	*Sargassum ilicifolium*	Rostami (Iran)	Fall (October) and winter (December)	MeOH extract	TPC	Fall: 363.69 ± 18.24 µg 100 g^−1^; Winter: <100 µg 100 g^−1^	[40]
Sargassaceae	*Sargassum tenerrimum*	Rostami (Iran)	Fall (October) and Winter (December)	MeOH extract	TPC	Fall: 308.81 ± 5.06 µg 100g^−1^; Winter: <100 µg 100g^−1^	[40]
Sargassaceae	*Sirophysalis trinodis*	Rostami (Iran)	Fall (October) and winter (December)	MeOH extract	TPC and TFC	TPC: 100 mg·100g^−1^; TFC: 1000 mg.100 g^−1^	[40]
Sargassaceae	*Colpomenia sinuosa*	Intertidal zone of the Qeshm Island, Persian Gulf (Iran)	Summer and winter 2016	MeOH extract	Pigments	Winter: 19.23 ± 0.0.65 µg g^−1^ DW; Summer: 13.53 ± 0.35 µg g^−1^ DW	[39]
Scytosiphonaceae	*Iyengaria stellata*	Intertidal zone of the Qeshm Island, Persian Gulf (Iran)	Summer and winter 2016	MeOH extract	Pigments	Winter: 55.39 ± 1.15 µg g^−1^ DW; Summer: 26.10 ± 0.85 µg g^−1^ DW	[39]

## Data Availability

No new data were created or analyzed in this study. Data sharing is not applicable to this article.

## Abbreviations

PC: phenolic content; GAE: gallic acid equivalent; EtOH: ethanol; H_2_O: water; DW: dry weight; Chl: chlorophyll; NR: unregistered; MDA: malondialdehyde; FW: fresh weight; TPC: total phenolic content; CE: catechin equivalent; MeOH: methanol; Hex: hexane; CHCl_3_: chloroform; DCM: dichloromethane; DMF: dimethylformamide; DMSO: dimethyl sulfoxide; PGE: phloroglucinol equivalents; LED: light-emitting diodes; UVA: ultraviolet A; UVB: ultraviolet B; UV: ultraviolet; L: light; D: dark; RGR: relative to growth rate; PAR: photosynthetically active radiation; W: watts; PES: Provasoli; PSII: photosystem II; EE: enriched experiment; NE: non-enriched experiment; VES: Von Stosch’s solution; NO_3_^−^: nitrate ion; CO_2_: carbon dioxide; N: nitrogen; Ae: aeration; NH_4_: ammonium ion; PO_4_: phosphate ion; CuSO_4_·5H_2_O: copper(II) sulfate pentahydrate; KNO_3_: potassium nitrate; KH_2_PO_4_: dihydrogen potassium; ppt: parts per trillion; DM: dry matter; EtAc: ethyl acetate; TFC: total flavonoids content, TTC: total tannins content; FRAP: ferric reduced power; C18:1n-9: oleic acid; DWE: dry weight extract; QE: quercetin equivalent; HPLC: high-performance liquid chromatography; TE: Trolox equivalents; DPPH: 2,2-diphenyl-1-picrylhydrazyl; ORAC: oxygen radical antioxidant capacity; RE: rutin equivalents; MUFA: monounsaturated fatty acids; PUFA: polyunsaturated fatty acids; UHPLC: ultra-high-performance liquid chromatography; UPLC: ultra-performance liquid chromatography; PDA: photodiode array; ESI: electrospray ionization; QTOF: quadrupole time-of-flight.
